# Playing Pokemon Go: Increased Life Satisfaction Through More (Positive) Social Interactions

**DOI:** 10.3389/fspor.2022.903848

**Published:** 2022-07-08

**Authors:** Tanja S. H. Wingenbach, Yossi Zana

**Affiliations:** ^1^Social and Cognitive Neuroscience Laboratory, Centre for Biological and Health Sciences, Mackenzie Presbyterian University, São Paulo, Brazil; ^2^Department of Consultation-Liaison Psychiatry and Psychosomatic Medicine, University of Zurich/University Hospital Zurich, Zurich, Switzerland; ^3^School of Human Sciences, Institute for Lifecourse Development, University of Greenwich, London, United Kingdom; ^4^Centre for Mathematics, Computation and Cognition, Federal University of ABC, Sao Bernardo do Campo, Brazil

**Keywords:** social interaction, lifestyle, life satisfaction, gaming, mental disorders, Pokemon Go, well-being

## Abstract

Pokemon Go (PoGo) is a social mobile game requiring both physical activity and social interaction, and previous research has reported positive effects of PoGo on physical health. However, little research has been conducted on the effects of PoGo on social functioning and life satisfaction, which are important factors for good mental health. The current study investigated the effects of PoGo on life satisfaction and social functioning in participants with and without self-reported diagnoses of mental disorders. Participants were 434 current PoGo players aged 18–69 of diverse genders and nationalities, with a subsample (*N* = 138) self-reporting diagnoses of various mental disorders with impairments in social functioning. Participants provided retrospective and current self-report measures about their PoGo use, life satisfaction, social functioning (sociality and social ability) and clinical symptom severity. Results showed higher self-reported social functioning and life satisfaction since playing PoGo compared to the time period before playing, which involved a shift from negative to positive ratings. The increases in self-reported life satisfaction and sociality (but not social ability) were more pronounced for the clinical compared to the non-clinical subsample. Results also showed the effect of the social ability change on the life satisfaction change was mediated by the sociality change and moderated by the number of daily in-person player interactions (including strangers). The findings here, using subjective judgements, show that PoGo motivates social interactions and increases life satisfaction, demonstrating that social mobile gaming provides an easy to implement tool to subjectively improve social functioning. This has important implications for populations with social difficulties and reduced social motivation.

## Introduction

A sedentary lifestyle can have detrimental effects on physical health of adults and children (meta-analyses by, e.g., Tremblay et al., 2011; Edwardson et al., 2012; Wilmot et al., 2012; Biswas et al., 2015) and social isolation can be harmful to their mental health (Matthews et al., 2015; Beutel et al., 2017). As such, being active and having social interactions can minimise the risk for many physical and mental disorders (e.g., Salmon, 2001; Mason and Holt, 2012). Though, while this is common knowledge, it is often difficult for people to find the motivation to engage in active leisure time activities or seek for people to socialise with. Many health interventions require additional efforts (e.g., going to meetings, accessing online reading materials) or pre-existing motivation for change to be successful, creating a hurdle, as shown by a web-based intervention to promote physical activity (see Hansen et al., 2012). Alternatively, things that are anyway integrated into people's life and routine could be considered to circumvent the hurdle. That is, to promote and facilitate movement and socialising, smartphones and commercialised Apps could be used.

Pokemon Go (PoGo) is a commercialised App for smartphones (see Supplementary Materials I for information on the game) and is a very popular social mobile game. Statistics of active monthly PoGo players show a range between 60 and 80 million people for all of 2020 up to March 2022 (according to activeplayer.io) and numbers surpassing 100 million in 2019. The reasons for its popularity might be its hybrid reality game nature where the physical space becomes one with the digital space making movement outside the home necessary and creating social encounters (see De Souza e Silva, 2017). These side-effects of playing Pogo align with the overarching aims of the game developer to encourage “movement, exploration, and face-to-face social interaction” (https://nianticlabs.com/).

The movement aspect of PoGo quickly attracted immediate attention from the research community with many published studies showing a decrease in sedentary behaviour (e.g., Althoff et al., 2016; Nigg et al., 2017) or a significant increase in the number of daily steps (Khamzina et al., 2020). While early PoGO research has shown that the game promotes and increases social interactions (e.g., Paasovaara et al., 2017; Rauschnabel et al., 2017; Zach and Tussyadiah, 2017), many of the game's features encouraging social interaction were added later to the game (see Supplementary Materials I). Importantly, it is possible that the main catalyst for continued gameplay *is* the social factor (rather than the game itself; see also Sobel et al., 2017) making it necessary for research to focus on social factors in relation to PoGo. A study aimed at identifying motives for playing PoGo indeed discovered two social factors among seven motives, friendship maintenance and relationship initiation; the other motives were exercise, fun, escapism, nostalgia, and achievement (Yang and Liu, 2017). Friendship maintenance was used as term by the authors to describe using PoGo as an activity to carry out with existing friends whereas relationship initiation referred to expanding the social circle by meeting new people. An 8-weeks intervention study in adolescents corroborates the latter in that 52% individuals of the experimental group (i.e., playing PoGo) reported that PoGo helped them make friends (Ruiz-Ariza et al., 2018). Interestingly, the PoGo group had significantly higher self-report ratings of social interaction ability after playing the game for 8 weeks compared to the non-playing control group (alongside better selective attention and concentration) while there were no group differences in the initial assessment (Ruiz-Ariza et al., 2018). It seems like playing PoGo not only requires social interaction to achieve the in-game goals but offers an opportunity to initiate new friendships and/or maintain existing bonds through which the confidence in one's own social interaction abilities can increase.

Given the social nature of humans and the importance of social interactions for wellbeing and mental health, the social aspects of PoGo might have more far-reaching positive effects. According to a general population-based study including 25,190 Italians (aged 18–64), the frequency of contact with friends and family as well as the quality of these social contacts, alongside social integration, and an active lifestyle are significant predictors of life satisfaction (Amati et al., 2018). It is noteworthy that these predictors align with the PoGo aims of movement and social interaction. Playing PoGo could positively affect life satisfaction. In line with this speculation, the motive of friendship maintenance for playing PoGo was found to be positively associated with life satisfaction (Yang and Liu, 2017). Moreover, a recent diary study on playing PoGo found the amount of social interactions and life satisfaction ratings to fluctuate in line with each player's daily playing time over a week (Ewell et al., 2020). The question arises whether playing PoGo can augment the quantity and quality of social interactions which, in turn, could enhance life satisfaction.

While many features of PoGo require social interaction, much of the game can still be enjoyed in solitude and players can choose to play alone or with others. The question arises whether people who prefer to and mainly do play alone benefit from playing PoGo as do players who prefer and mainly engage in group play. To our knowledge, no study has investigated playing with others or alone and preferring to play alone vs. with others in relation to the positive effects reported by PoGo players.

As a *social* mobile game, playing PoGo might even benefit individuals with social insecurities. This is because PoGo has been found to create a sense of community in players (Zach and Tussyadiah, 2017) and it was pointed out that becoming a member of a community with shared interests (here: PoGo) can help overcome social insecurities (Tabacchi et al., 2017). In addition, a pilot study reported that 40% of their PoGo playing general population participants stated to have experienced a reduction in anxiety about leaving the house, exploring new places, and engaging with strangers since playing the game (Kogan et al., 2017). Many mental disorders are associated with social isolation or difficulties in social interactions. For example, main characteristics of Major Depression are loss of interest and reduced activity (see diagnostic criteria: DSM-V; American Psychiatric Association, 2013) and impairments in social functioning are very common (literature review by Hirschfeld et al., 2000). Data from the large Netherlands Study of Depression and Anxiety (i.e., the two most common mental disorders) showed that mood and anxiety disorders are strongly associated with impaired social functioning (Saris et al., 2017). An activity that is easy to carry out while encouraging (positive) social interaction might be particularly helpful to people with symptoms of mood and anxiety disorders. Facilitation of social interaction and societal inclusion are also of importance for individuals with high-functioning Autism-Spectrum-Conditions (ASC) where social impairments are central (American Psychiatric Association, 2013). Benefits on psychosocial functioning and life satisfaction from playing PoGo might be observed in the general population including sub-clinical and clinical populations with mental disorders. Indeed, a recent study found depression-related internet searches across 12 countries to be markedly reduced in the respective countries after PoGo release compared to before (Cheng et al., 2022). It should be noted that internet searches are indicative of individuals' interest in depression-related information and are not a direct measure of depression. To date, no published research exists (to our knowledge) on potential benefits of playing PoGo for clinical populations.

**Figure 1:**
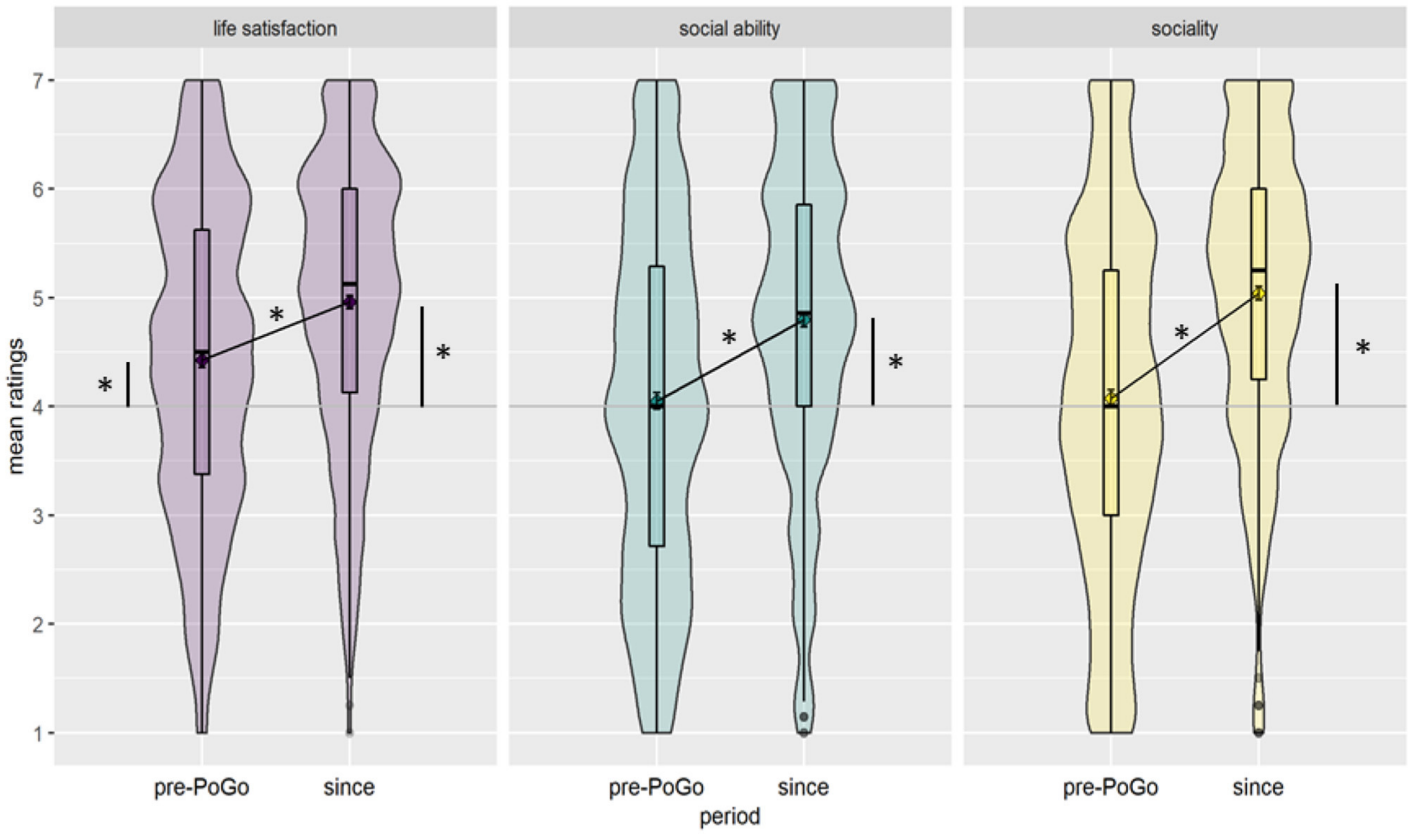
Ratings of perceived life satisfaction, social ability, and sociality. The ratings for the time period before playing PoGo (pre-PoGO) and since playing PoGo (since) are shown. Data distributions are shown by the violin plots. The rhombus within the boxplots represents the sample mean while the black horizontal line represents the median. The grey line at the value 4 represents the middle of the rating scale, i.e., neutral rating, while all ratings above 4 represent positive and below 4 negative perceptions. Error bars represent 95% confidence intervals of the means. The diagonal lines show the significant comparisons between the time periods; the vertical lines show the significant comparisons to the midline of the rating scale. *p < 0.001.

The current study aimed to examine perceived psychosocial effects of the social mobile game PoGo on its players from the global adult general population. To this end, players were asked within a survey to report about their lifestyle, life satisfaction, and social functioning both for the period before and since they started playing PoGo. It was hypothesised that, in general, PoGo has overall positive effects on players. Several hypotheses were specifically tested:

1. Playing PoGo positively affects social functioning and life satisfaction.
2. A greater increase in social functioning from the time period before to since playing PoGo predicts a greater increase in life satisfaction, with the quantity of daily interactions having a moderating role (see Supplementary Materials II, Figure 1, for visualisation).
3. Social and solitude players perceive an increase in social functioning and life satisfaction.
4. Players with self-reported diagnoses of mental disorders perceive greater changes than those without, and depression and trait anxiety levels, as well as autism-like traits are positively associated with changes in social functioning and life satisfaction.

## Methods

### Participants

Recruitment took place on various online platforms and social media (e.g., Facebook, WhatsApp, Discord, Telegram, and reddit) from April to August 2019; the sample size (*N* = 503) was determined by the number of participants who filled out the survey within this time frame. Due to a continuous introduction of additional game features and thus gaming experience, the period of data collection was restricted. There were versions of the survey in Brazilian Portuguese, German, and English; 229, 208, and 66 individuals filled out the versions, respectively. Inclusion criteria were a minimum age of 18 years and actively playing PoGo (at any frequency). Sixty-nine participants were excluded as they stated an age lower than 18 years, not stated their date of birth, or entered impossible dates, e.g., such in the future. All participants were PoGo players the game at the time of filling out the survey. Thus, the sample size for statistical analyses was 434, composed of 204, 172, and 58 individuals from the Portuguese, English, and German version, respectively. The mean age of the sample was 31 years (*SD* = 9.5), ranging from 18 to 69 years, and consisted of 285 males and 144 females; additionally, 5 individuals self-identified as agender or non-binary.

The percentage of self-reported biologically male and female participants in each version of the survey was 75/25% in the Brazilian Portuguese one, 61/36% in the English one, and 47/53% in the German one. These differences were significant, χ^2^ = 17.94, *p* < .001. Thus, additional analyses were conducted on sex differences and cultural differences regarding the DVs of the current study. Most players reported to have mainly engaged in indoor hobbies prior to playing PoGo (*n* = 322) with *n* = 18 having reported outdoor hobbies and *n* = 42 having reported both types of hobbies. *N* = 90 participants self-reported a formal diagnosis of a mental disorder (Table 1). See Supplementary Materials II for further participant characteristics, such as employment status, marital status, trainer level, playing time etc.

**Table 1:** Self-reported formal diagnoses of a mental disorder.

**Diagnoses**	***n***
Depression	57
Anxiety	26
Attention deficit (hyperactivity) disorder	21
Autism spectrum disorder	11
Obsessive compulsive disorder	5
Bipolar disorder	4
Borderline personality disorder	4
Panic disorder	4
Post-traumatic stress disorder	4
Generalised anxiety disorder	3
Social phobia	3
Trauma	1
Dissociative disorder	1
Burnout	1
Insomnia	1
Dyslexia	1
Linguistic disorder	1
Binge eating	1

*Some participants reported multiple diagnoses of mental disorders*.

### Measures

The survey included questions on demographic characteristics, mental health, game-specific questions, direct judgement on the effect of the game on participants, and questions on life satisfaction and social functioning; see Supplementary Materials II and Supplementary Table S1-II, for more detail. Most questions were presented in forced choice format, and some had multiple response options; few questions had an open response format-indicated in the Supplementary Materials. The life satisfaction items were taken from the validated life satisfaction scale (Diener et al., 1985), which consists of five items, but was extended by three items formulated by us. Three items assessing social functioning were taken from Zach and Tussyadiah (2017) and slightly reworded and 10 items created by us were added. The items on life satisfaction and social functioning were worded to once address the time before playing PoGo and once addressing the time since playing PoGo. The answering format these items was a 7-point Likert-scale ranging from 1 = “completely disagree” to 7 = “completely agree”.

The survey further included validated questionnaires that are typically used in clinical settings assessing symptom severity. The Beck's Depression Inventory (BDI; Beck et al., 1996), the state items of the State-Trait Anxiety Inventory (STAI; Spielberger et al., 1983), and the Autism-Quotient (AQ; Baron-Cohen et al., 2001) were used. The published German (STAI: Laux et al., 1981; BDI: Drieling et al., 2007) and Portuguese versions (BDI and STAI: Gorenstein and Andrade, 1996) of the three questionnaires were used; German translation of the Autism Quotient (AQ) by Juergen Kremer, Portuguese translation of the AQ by Ana Osorio and Beatriz Sanchez.

### Procedure

Participants filled out the survey on-line through Google forms. Consent for participation was obtained from a first form, which included information on the study titled “Effects of playing PoGo on everyday life”. That is, participants were informed that “participation in this study takes place on-line and entails questions about your socio-economic status, habits of playing PoGo, life satisfaction, and social interaction”. Participants had to sign the consent form by typing in their name and were sent a copy of the consent form to the email address they provided there. After the consent form was submitted, individuals were provided with a link to the actual survey. This approach assured anonymity, as participants' names were kept separate from the data.

Participants were asked to think about the time *before* they started to play PoGo to fill out the sections of life satisfaction and social functioning. Participants were not informed at this point that they would have to make the same judgements about the time *since* playing PoGo. This approach was chosen to minimise biassing the answers.

After the assessment of social functioning and life satisfaction, the option was provided to either submit the survey or to continue with the clinical questionnaires with the option to submit after each of the added questionnaires or to continue. This approach was chosen to minimise data loss of participants who did not want to invest the time needed to fill out the complete survey (~30 min), since only submitted answers are stored on Google forms.

Participants were provided with the opportunity to leave a comment; the text box allowed to let the researchers involved in the study know if anything was not asked for, they feel like should have been asked. After submitting the survey, the option was provided to type in an email address to be considered for a prize draw, which was a Play Store or iStore voucher allowing participants to purchase in-game currency. One voucher was given away per 100 participants for $10 in the currency of the winners. The prize draw was conducted using an online random number generator. However, Brazilian participants were excluded based on the country's regulations prohibiting research participant compensation.

### Data Preparation and Statistical Analyses

Response re-coding and categorisation are explained in the Supplementary Materials II. An exploratory factor analysis was conducted on the re-coded items assessing participants' life satisfaction and social functioning, once for the items relating to the time before and once for the time since playing PoGo (see Supplementary Materials II for details). These analyses aimed at checking whether the self-formulated items would fall into the categories of life satisfaction and social functioning to be able to conduct the hypotheses testing with these factors. A 3-factor structure was found in both pre-game and since playing analyses, explaining 68% and 69% of the variance, respectively: “Life satisfaction” and social functioning was split into “Sociality” and “Social ability”. The factor “Sociality” contained items addressing the quantity and quality of participants' social interactions. The factor “Social ability” contained items addressing participants' abilities in socialising (see Supplementary Materials II and Supplementary Table S2-II). The items belonging to each of the three identified factors were combined and means were calculated across items per participant both for the pre-game period and since playing the game; those means were used for the statistical analyses.

Data analyses were conducted in IBM SPSS, Version 25.0. Figures were created in R (R Studio Team, 2019) using the package ggplot2 (Wickham, 2016). Statistical comparisons were conducted 1-tailed for testing directed and 2-tailed for undirected hypotheses which is also indicated in the results in the Supplementary Materials III. Bonferroni-Holm correction of the *p*-values (Holm, 1979) was always applied when more than one comparison on the same variable was conducted to account for multiple testing; reported *p*-values are after correction.

## Results^1^ and Discussion

### Effects of PoGo on Players' Social Functioning and Life Satisfaction (Hypothesis 1)

Participants reported higher *life satisfaction, social ability and sociality* for the period since playing the game compared to the pre-game period (Figure 1), in line with the hypothesis. These results also align with previously published PoGo studies on life satisfaction (Yang and Liu, 2017; Ewell et al., 2020) and social ability (Ruiz-Ariza et al., 2018). The increased sociality reports from the current study can be explained by the game design and the behaviours it enforces. According to De Souza e Silva (2017), games like PoGo are social activities and create social connectedness while the overlay of gameboard and physical world allows to flexibly switch back and forth between the two. Consequently, the game easily integrates in daily life and routines (Hjorth and Richardson, 2017), motivates going outside to play, and counters social isolation (Evans and Saker, 2019). How life satisfaction, social ability, and sociality are connected (in the context of playing PoGo) is discussed below under Hypothesis 2.

It is noteworthy that the current study found not only an increase in ratings of life satisfaction, social ability, and sociality but also a perceived change in quality. That is, players reported scores on the positive end of the rating scales for the playing period as opposed to neutral or negative ratings for the time before playing PoGo for life satisfaction, social ability, and sociality. At least on a subjective retrospective level, players perceive PoGo to positively affect their life on factors highly relevant for good mental health. (For effects of PoGo on players' lifestyle and effects of different playing motivations on the change in social functioning/life satisfaction, see Supplementary Materials III.)

The current study considered potential results-influencing factors by conducting additional analyses on age and “culture”. Results showed increases in ratings on life satisfaction, social ability, and sociality for males and females and for Northern Americans, Southern Americans, and Europeans. Further, males and females reported similar judgements for the period since playing the game (see Supplementary Materials; Supplementary Figure S1-III). There were no marked differences between Northern Americans, Southern Americans, and Europeans in ratings for the two time periods as indicated by non-significant interactions (see Supplementary Materials III for statistics and Supplementary Figure S2-III). It seems that the perceived effects of playing PoGo on social functioning and life satisfaction are independent of sex and “culture”/playing location, at least for the western world.

There are positive associations between PoGo gameplay (i.e., number of playing days/hours) and the experience of positive emotions and friendship initiation and intensification (Bonus et al., 2017), which could explain the increase in social functioning in life satisfaction in the current study. One could argue that it would be necessary to allocate many hours to the game each day to experience the aforementioned benefits, which could lead to addiction and associated problems. A study reported that PoGo gameplay intensity measured daily (over the course of 1 week) was positively associated with the amount of social interactions and life satisfaction players reported while, noteworthy, there was no association between the mean playing time across the week and life satisfaction (Ewell et al., 2020). That is, the intra-individual playing time had a greater effect on players than the inter-individual playing time. In the current study, the daily playing hours were also positively associated with the amount of daily face-to-face player interactions (*r* = .27, *p* < .001) with a small positive association with the change score in life satisfaction (*r* = .12, *p* = .014). The mean playing days per week reported was 6.57 (*SD* = 1.04) with a mean of 2.78 h per day (*SD* = 1.89). These results combined suggest that excessive gameplay is unlikely necessary for experiencing the positive effects of playing PoGo. In fact, Ewell et al. (2020) speculated that the quantity of daily social interactions contributes to greater life satisfaction ratings, which is part of the model that was tested in the current study (next section).

### Model Predicting Changes in Life Satisfaction (Hypothesis 2)

The posed model assumed that increases in social functioning from the time period before to since playing PoGo predict increases in life satisfaction while considering a moderating role of the quantity of daily interactions players have amongst each other. Results from linear regression analyses identified face-to-face interactions with strangers and friends as stronger predictor than interactions with friends only whether in-person or in-game. Thus, identified face-to-face interactions with strangers and friends were entered in the model. Results from the model test showed that with increasing change in social ability, the changes in sociality and also in life satisfaction increased; the change in life satisfaction through sociality was amplified by a higher quantity of face-to-face player interactions; Table 2. (The conditional effect of change in sociality on life satisfaction as moderated by the amount of player interactions is visualised in Supplementary Figure S3-III.) The significant moderated mediation model suggests that the more players perceived their social ability to have improved since playing PoGo, the more the quality and quantity of their social interactions increased, which, in turn, increased their life satisfaction. The larger the number of face-to-face players encounters, the greater was the effect on life satisfaction. These results highlight the importance of the social factors but are in slight contrast to previous research where a model was tested and found no effect of the social factors friendship initiation and intensification on wellbeing in PoGo players (Bonus et al., 2017). The advantage of the current study, however, is that two time periods were assessed allowing to calculate change scores rather than obtaining an assessment for one (current) time period. In addition, the current study showed that the social factors involved in playing PoGo to positively affect life satisfaction can also involve interactions with strangers not to the degree of friendship initiation and intensification. The current study's findings aligns with a study reporting facilitated interactions with strangers due to playing PoGo (Vella et al., 2019). Vella et al. (2019) stated having to go outside and a shared interest in the game as reasons for facilitated social interactions and creating a sense of belonging. The findings from the current study align further with an Italian general population-based study (Amati et al., 2018), which included participants of a very similar age range as in the current study. Amati et al. (2018) reported predictors of life satisfaction that are positive side-effects of PoGo, i.e., increased quantity and quality of social interactions, movement, and a sense of community/integration. Combined with the current study, such findings highlight the importance of social interactions for humans, whether with friends or strangers, and how they essentially contribute to life satisfaction.

**Table 2:** Moderated mediation model predicting the change in life satisfaction.

**Effect**	**Model summary**	
X on M	Social ability change (X) on sociality change (M)	*b* = 1.00, *SE* = 0.05, 95% *CI* (0.91, 1.09), *t*_(432)_ = 21.87, *p* < 0.001
Constant		*b* = 0.22, *SE* = 0.06, 95% *CI* (0.10, 0.34)
	*F*_(1, 432)_ = 478.48, *p* < 0.001, *r*^2^ = 0.53	
X on Y	Social ability change (X) on life satisfaction change (Y)	*b* = 0.25, *SE* = 0.05, 95% *CI* (0.16, 0.35), *t*_(429)_ = 5.16, *p* < 0.001
M on Y	Sociality change (M) on life satisfaction change (Y)	*b* = 0.16, *SE* = 0.04, 95% *CI* (0.08, 0.24), *t*_(429)_ = 4.02, *p* < 0.001
W on Y	Quantity of player interactions (W) on life satisfaction change	*b* = −0.004, *SE* = 0.01, 95% *CI* (−0.02, 0.10), *t*_(429)_ = −0.56, *p* = 0.575
M^*^W on Y	Sociality change (M)^*^quantity of player interactions (W) on life satisfaction change (Y)	*b* = 0.01, *SE* = 0.00, 95% *CI* (0.04, 0.27), *t*_(429)_ = 2.64, *p* = 0.009
Constant		*b* = 0.16, *SE* = 0.06, 95% *CI* (0.10, 0.34)
	*F*_(4, 429)_ = 58.70, *p* < 0.001, *r*^2^ = 0.35	
Moderated mediation		index^*^= 0.01, *SE* = 0.00, 95% *CI* (0.001, 0.013)

*Model number 14 was used*.

* *Index = index of moderated mediation (Hayes, 2015)*.

### PoGo Social Play Habits and Preferences (Hypothesis 3)

Despite the many features of PoGo that promote social interaction, the game can be successfully played alone, which led to examine the effects on life satisfaction and social functioning considering social vs. non-social playing preferences and habits with the current study (the distinction between preference and behaviour was made, since circumstances do not always allow to behave in line with one's preferences). For both social and solitude players, the results regarding life satisfaction, social ability, and sociality were in line with the hypothesised positive effect of playing PoGo. Although the change in score was highest for the participants who reported to prefer to play in groups but to mainly play alone, there was an increase in perceived life satisfaction, social ability, and sociality from the pre-game period to since playing PoGo in *all* groups (see Supplementary Figures S4-III–S6-III; Supplementary Tables S2-III–S5-III in Supplementary Materials III). The findings suggest that most individuals, and especially those who crave social interaction, can positively affect their life satisfaction through social interactions. This assumption aligns with findings from a large general population study which found that life satisfaction increases with increasing expected or experienced positive social interactions (Lewinsohn et al., 1991). These results underpin that the social aspects of PoGo can be beneficial not only for social functioning but also for life satisfaction. (For effects of social vs. solitude game feature use on the change in social functioning/life satisfaction, see Supplementary Materials III.)

A preference of solitude can be tied to a person's personality. Research on early players of PoGo found many players to score high on introversion (Tabacchi et al., 2017) and highly introverted individuals do not seek out social interactions to the extent extroverts do. Interestingly, Hills and Argyle (2001) found that extroverts as well as introverts can gain happiness and satisfaction from social interactions, the difference being that extroverts interact with a larger number of people than introverts and introverts interact more with few close individuals. PoGo is a suitable leisure time activity for individuals who seek to socialise with many as well as with few individuals, which can contribute to a perceived increase in life satisfaction.

Interestingly, the groups differed from each other on their social ability ratings only for the period since playing PoGo and not before. Social players showed greater increases in social ability than solitude players. The results suggest that the social aspect of the game contributes to players' confidence in their social interaction abilities. A possible explanation is provided by the Social Cognitive Theory (Bandura, 1986) positing that psychosocial functioning can be explained by an individual's experiences with the environment (especially interactions with others) and accompanying cognitive evaluations. That is, repeated positive social interaction (as from playing PoGo) can strengthen people's confidence in their social behaviour (see also discussion below on mental health) as seen in increased social ability ratings in all groups for the playing period and amplified for social players who naturally have more social interactions.

In contrast, differences in sociality ratings, i.e., the quantity and quality of social interactions, were evident between the groups at both time periods. It is understandable that social people, also without playing PoGo, will have a greater amount of social interactions than people who are less social. A preference for solitude has the consequence that fewer social interactions are experienced and also offers less opportunities for encountering positive social interactions (Pavot et al., 1990). The consequences of the preferences could explain the group differences in sociality at both time periods. Participants who mainly play alone benefitted in terms of sociality from playing PoGo nonetheless, possibly because the game induces social interactions despite a preference for solitude.

Results from a study investigating the effects of interactions with strangers on people's self-reported affect compared to a solitude condition help further explain why also the solitude preferring PoGo players experienced positive changes. This experimental study found positive effects on affect across various experiments of interactions between strangers (Epley and Schroeder, 2014). That is, even if people think they prefer not to interact with others (as is a common regarding strangers), the experience is likely more positive than when opting for solitude. The results by Epley and Schroeder (2014) were similar regardless of whether people were instructed to talk to someone or were talked to by someone. This is in line with another study where participants were asked to either behave talkative or reserved and participants overestimated the amount of negative affect they would experience from acting talkative if that was not their nature (Zelenski et al., 2013). These two studies indicate that positive experiences can emerge also for more reserved individuals. Though, the benefit does not only apply to interactions with strangers but also with acquaintances and friends, which leisure time activities often involve. An investigation on motivations for a range of leisure time activities found the opportunity for social interaction to be a significant predictor for the vast majority of the activities included in the study (Hills et al., 2000). Compared to the other motivators (skill, challenge, purpose, and ability), the social motivator was strongest (Hills et al., 2000). The latter study suggests that it is not only the enjoyment of the activity itself that motivates individuals to engage in the activity but also the social interactions it creates. Combining the results presented here, social interaction is a need that can be satisfied within leisure time activities and social interactions (e.g., as created by playing PoGo) can be enjoyable positive experiences also for individuals with less need for social interactions.

### PoGo and Mental Health (Hypothesis 4)

As would be expected, participants who self-reported to have a formal diagnosis of a mental disorder (diagnosis group) had lower scores on the three factors (life satisfaction, sociality and social ability) than those who did not report such a diagnosis. However, for the diagnosis group, the ratings from before playing PoGo were (descriptively) below the midpoint of the scale (i.e., negative) whereas the ratings since playing the game were above the midpoint of the scale (i.e., positive; Table 3).

**Table 3:** Means and SDs for the three factors by time period and diagnosis group.

**Factor**	Diagnosis^*^	**Pre-PoGo**	**Since-PoGo**	**Difference**
		***M SD***	***M SD***	**(since-pre)**
Life satisfaction	No	4.72 ± 1.35	5.18 ± 1.21	0.46^a^
	Yes	3.42 ± 1.22	4.22 ± 1.30	0.80^a^
Social ability	No	4.21 ± 1.56	4.91 ± 1.40	0.70
	Yes	3.53 ± 1.68	4.46 ± 1.58	0.93
Sociality	No	4.26 ± 1.60	5.13 ± 1.30	0.87^b^
	Yes	3.46 ± 1.44	4.77 ± 1.45	1.31^b^

* *The group with self-reported diagnoses had significantly lower means on life satisfaction, social ability, and sociality for both periods before and since playing the game than the group without self-reported diagnoses*.

a, b *Group comparisons on the change scores per factor showed significant differences with greater changes for the diagnosis group. The respective statistical results are presented in the Supplementary Materials III*.

In line with the hypothesis, results showed that participants who self-reported to have a formal diagnosis of a mental disorder had a significantly higher positive change in life satisfaction and sociality than those who reported not having such a diagnosis. However, the group differences for the change in social ability showed a trend towards statistical significance with the groups' ratings nearing each other for the playing period. Results also showed, the greater the depression levels and trait anxiety levels (but not autism-like traits), the greater the increase in sociality. These two correlations were small but positive (see Supplementary Materials III and Supplementary Figure S7-III). There were no significant correlations between the AQ, BDI, and TAI scores and the change in life satisfaction or social ability.

That the current study found perceived social functioning and life satisfaction to be lower in the diagnosis group than the no-diagnosis group and sociality to be negatively associated with symptomatology is in line with the known effects mental disorders can have on people. The current study thus aligns with the definition of mental disorders (American Psychiatric Association, 2013) that individuals with symptoms/diagnoses of mental disorders perceive impairments in life satisfaction and social functioning. However, worth noting is that the ratings on all three factors were negative (i.e., below the neutral midpoint of the scale) for the period before playing PoGo in the diagnoses group and reached the positive side of the scale for the period since playing PoGo. That is, not only did those participants perceive an improvement in life satisfaction and social functioning since playing PoGo but also reached a positive outlook. The current results on PoGo and mental health align with the wider published literature. For example, Wang (2021) conducted a literature review on the topic and found 19 out of 25 published studies to report positive effects on mental health related to playing PoGo. The current study further aligns with the findings by Cheng et al. (2022) of depression-related internet searches to have decreased after PoGo release in the respective country by applying a similar time period comparison as in the current study. Importantly, the current study extends Cheng et al. (2022) by explicitly including individuals with self-reported sub-clinically and clinically relevant levels of symptomatology of mental disorders. In addition, it is particularly noteworthy that players with a self-reported diagnosis of a mental disorder perceived greater changes in life satisfaction and sociality than those without a self-reported diagnosis. These findings can, to some degree, be explained by generally lower ratings by the diagnosis group to start with, leaving more room for improvement. It can be assumed, though, that the driving mechanisms are similar in both groups.

The just presented results (from categorical and dimensional analyses) suggest that engaging in a leisure time activity such as PoGo can affect factors that can contribute to mental health (e.g., physical activity and social interaction). A recent qualitative study on the effects of video game play on mental health and behavioural problems in recovering veterans found that the effects were overall positive (Colder Carras et al., 2018). The veterans stated that gaming provided them with connection, meaning, and distraction, although they sometimes felt isolated as well. For one, the effects overlap greatly with the benefits the current study found. For another, PoGo has the potential to overcome experienced isolation by bringing people together face-to-face. Bonus et al. (2017) found that the increase in exercise due to PoGo was associated with a decrease in depression. Cheng et al. (2022) further investigated potential underlying mechanisms for the aforementioned decrease in depression-related internet searches and identified physical activity, opportunities for social interaction, and interaction with green spaces as modifying factors. Together, it seems as though playing PoGo changes behaviours associated with depression to behaviours associated with mental health, as playing PoGo requires physical activity and offers opportunities for social interaction by meeting other players at playing hotspots. In addition to meeting other PoGo players at gaming hotspots, sharing the same hobby, i.e., playing PoGo, provides game-related topics for conversation, which can facilitate approaching other players and make positive social interactions more likely. A highly influential psychological theory (Social Cognitive Theory) postulates that the repeated experience of positive social interactions can reinforce seeking more social interactions (Bandura, 1986). In addition, the behaviour of socially competent individuals can be observed and imitated (Bandura, 1986). Any form of facilitation of socially apt behaviour is particularly helpful to people with social insecurities or impaired social functioning. Positive social interactions can increase an individual's belief in their social abilities, which, in turn, can increase their contact initiation, and both can increase life satisfaction.

## Summary and Limitations

The current study investigated perceived effects PoGo can have on player's social functioning and life satisfaction. PoGo seems to motivate people who would normally engage in indoor and solitude leisure time activities to go outside, be physically active, and to seek out companionship of other players while it does not seem to substitute pre-existing hobbies or to interfere with important things in life. Consequently, playing PoGo can introduce healthy habits to people's lifestyles. The current study had participants reflect on their social functioning and life satisfaction before and since playing PoGo and the ratings from both time periods were statistically compared. Reported social functioning and life satisfaction were greater and more positive for the period since playing the game than before. Importantly, these findings were robust also when considering potential hampering factors such as a preference for solitude. The strength of the effects was modulated by playing motivation, preference and habit of playing alone or with others, and diagnosis of a mental disorder. Additional analyses showed robustness of the findings across the factors “culture” (continent) and sex. Moreover, the current study postulated a model and found an increase in social ability to predict an increase in the quality and quantity of social interactions which, in turn, predicted an increase in life satisfaction while the amount of daily face-to-face social interactions with other players moderated the latter effect. This study suggests that PoGo players may improve their life satisfaction through social interactions, corroborating findings that life satisfaction increases with increasing expected or experienced positive social interactions (Lewinsohn et al., 1991). Overall, the results from the current study suggest that the game might serve as a tool to implicitly (in a playful way) increase individuals' satisfaction with their social life, relationships, and life in general.

However, the instructions focussed more on time periods than on the game itself. It is possible that also other factors in life beyond the game might have affected people's increase in life satisfaction and social functioning, although the results from the “direct items” support an effect of the game itself. Nonetheless, the current study design does not allow for drawing causal inferences. It was planned to carry out a follow up study with new players 3 months after the initial participation to overcome the limitation of retrospective judgements participants were asked to make in the current study and to be able to causally test the effect of playing PoGo on life satisfaction and social functioning. However, this could not be conducted due to a lack of new players in the sample. That is, most participants have been playing PoGo for several years and so the effect of playing PoGo on players could not be investigated beyond what is presented in this manuscript. Nonetheless, the results suggest that engaging in a leisure time activity, such as PoGo, can affect factors related to mental health and, together with results from previous studies, indicate that incorporation of PoGo in therapy like cognitive-behavioural therapy should be empirically investigated within future research.

## Data Availability Statement

The datasets presented in this article are not readily available because the consent form signed by participants stated that the data will be accessible only to project members and deleted after 5 years.

## Ethics Statement

The studies involving human participants were reviewed and approved by Research Ethics Committee at the Federal University of ABC (3.216.797). The patients/participants provided their written informed consent to participate in this study.

## Author Contributions

TW and YZ conceptualised and designed the study. TW organised the database, performed the statistical analysis, and wrote the first draft of the manuscript. YZ revised the manuscript. Both authors read and approved the submitted version.

## Conflict of Interest

The authors declare that the research was conducted in the absence of any commercial or financial relationships that could be construed as a potential conflict of interest.

## Publisher's Note

All claims expressed in this article are solely those of the authors and do not necessarily represent those of their affiliated organizations, or those of the publisher, the editors and the reviewers. Any product that may be evaluated in this article, or claim that may be made by its manufacturer, is not guaranteed or endorsed by the publisher.

## Footnotes

^1^See Supplementary Materials III for statistical analyses and detailed results.
