# Supplementary material for: Playing Pokemon Go: Increased Life Satisfaction Through More (Positive) Social Interactions
Source: Front Sports Act Living. 2022 Jul 8;4:903848. doi: 10.3389/fspor.2022.903848 (PMC9304577; doi:10.3389/fspor.2022.903848)
Supplement: Supplementary file 1 [file Data_Sheet_1.docx]

# **Playing Pokemon Go: Increased Life Satisfaction Through More (Positive) Social Interactions**

**Tanja S. H. Wingenbach & Yossi Zana**

# **Supplementary Materials I: PoGo Game Features**

The main goal of the game is to complete the Pokedex, i.e., to collect all Pokemon available in the game. Achieving this goal requires players to walk around in the real world and search for Pokemon as well as hatch them from eggs or acquire them from raids. The raid system requires players to coordinate themselves, as there is no in-game communication feature. It thus encourages social interaction through social media (like WhatsApp) as well as face-to-face when battling the raid boss. The social aspects of the game became even more apparent when a friendship system was added to the game. Each player has a unique code they can share with others and include them in their friends list. Daily interactions between those players (e.g., by sending gifts, raiding or battling gyms together) increase the friendship level which unlocks benefits (e.g., attack bonus in joint battles). Trading of Pokemon has also been added to the game and is linked to the friendship feature, since only friends can engage in trades and must be within 100m from each other during trades. Yet another social feature was added later to the game, player vs player battling (PvP). Players could scan each other’s codes to battle each other, which required people to interact with each other (note: it is now possible to challenge others remotely).

**Pokedex**: Catalogue containing all Pokemon released in PoGo.

**Pokestop**: Releases items, e.g., pokeballs to capture Pokemon with, potions to revive Pokemon after battle, eggs to hatch Pokemon, gifts to send to friends.

**Eggs**: Eggs can be incubated and a Pokemon will hatch after walking; requirements of 2km, 5km, 7km, or 10km. (It should be noted that since data collection was completed, 12km eggs were added to the game).

**Teams**: There are three teams in the game, Valor, Mystic, and Instinct. Each player chooses one team.

**Gym**: A gym can be battled if it is occupied by an opponent team. Once defeated, a Pokemon can be placed in the gym to defend.

**Raids**: Raids take place at random times where a very strong Pokemon takes over a gym with varying levels of difficulty and a group of players must gather to collectively defeat the raid boss of higher levels.

**Trading**: Players can trade Pokemon with each other. Some Pokemon are region-specific, i.e., only available in certain parts of the world, making them popular trading material.

**PvP**: Players can challenge each other by scanning a QR code (or the friends list). (It should be noted that the PvP feature has been adapted since data was collected for the current study and it is now possible to battle players around the world through the game without any social interaction or even knowing the opponent)
