# Supplementary material for: Playing Pokemon Go: Increased Life Satisfaction Through More (Positive) Social Interactions
Source: Front Sports Act Living. 2022 Jul 8;4:903848. doi: 10.3389/fspor.2022.903848 (PMC9304577; doi:10.3389/fspor.2022.903848)
Supplement: Supplementary file 2 [file Data_Sheet_2.docx]

# **Playing Pokemon Go: Increased Life Satisfaction Through More (Positive) Social Interactions**

**Tanja S. H. Wingenbach & Yossi Zana**

# **Supplementary Materials II: Methods**

Hypothesis 2 proposed that a greater increase in social functioning from the time period before to since playing PoGo predicts a greater increase in life satisfaction, with the quantity of daily interactions having a moderating role. After conducting a factor analysis, the factor social functioning was split in two factors, so the tested model was modified to represent these two factors; Figure S1-II.

**Figure S1-II**


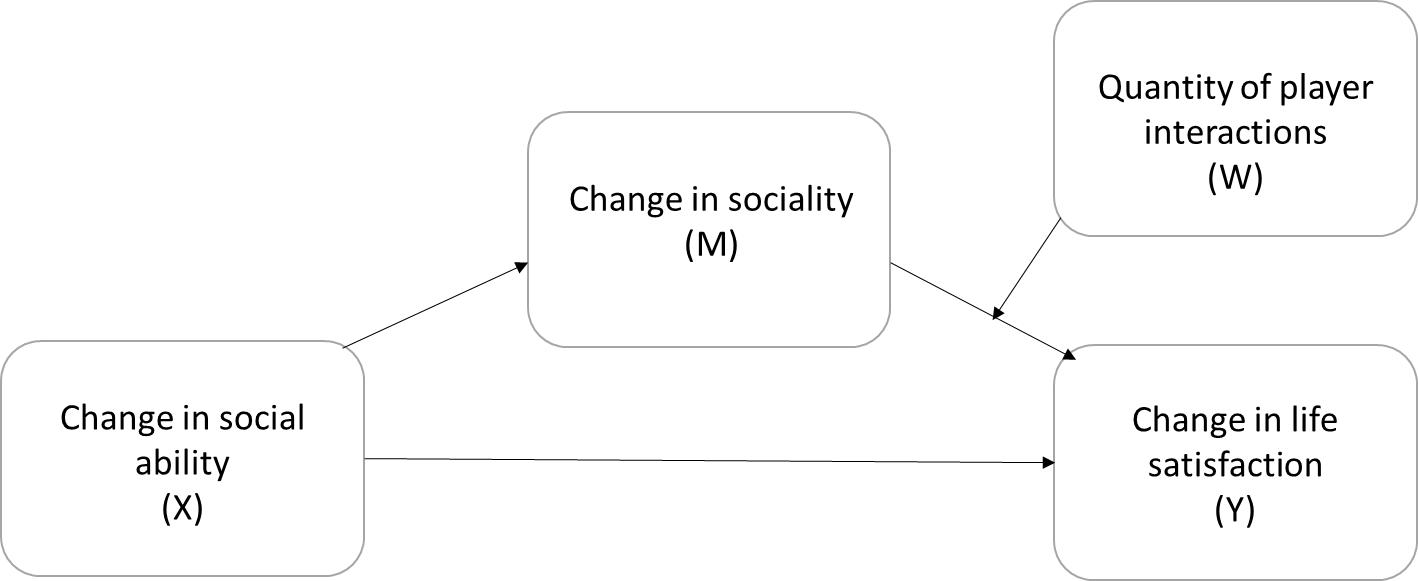
*Moderated Mediation Model (Hypothesis 2)*

*Note*. Moderated mediation model predicting the change in life satisfaction based on the change in sociality and considering the quantity of daily interactions with other players.

# **Participant Characteristics**

Most participants reported to be in employment (*n* = 265; 61%), 87 (20%) reported to be students, 37 (9%) reported to be self-employed while 3 (1%) people reported be have retired, and 42 (10%) reported to be unemployed. The majority of participants reported to be in a relationship without being married (*n* = 179), 150 reported to be single, 99 reported to be married, 2 to be widowed, and 3 to be divorced. Of the people who are in a relationship, 27 reported to have found their partner through playing PoGo. About 32% of the participants (*n* = 138) reported to have met most of their current friends through playing PoGo.

Most participants reported to have reached the maximum trainer level in the game (*n* = 234), which was 40 at the time of data collection and is currently 50, with a mean of 37.80 (*SD* = 3.56). Further, most participants started playing PoGo in 2016, the year the game was launched (*n* = 369), 30 reported a start in 2017, 17 in 2018, and 17 in 2019. (The starting date could not be determined for one person based on their response). Almost half of the participants played the game continuously since their starting date (*n* = 202) with the rest reporting to have taken a break at some point, which varied between 0.12 and 36 months (*Mdn* = 1.00 month). The mean playing days per week reported was 6.57 (*SD* = 1.04) with a mean of 2.78h per day (*SD* = 1.89). The vast majority of participants reported to play in urban settings (*n* = 391) and 41 reported to play rurally with 2 reporting to play mainly at home.

**Measures**

Table S1-II shows the survey sections and the respective measures per section.

**Table S1-II**

*Survey Sections and Measures*

| **Demographic characteristics** | sex^[[1]](#footnote-1)^*, date of birth, nationality*, living location*, profession*, employment status (employee vs self-employed vs unemployed vs student), marital status (single vs in a relationship vs married vs divorced vs widowed) |
| --- | --- |
| **Mental health** | diagnosis |
| **Game-specific questions** | trainer level*, starting date, breaks from playing (played continuously vs took a break, if so: duration*), experience points*, team (valor vs mystic vs instinct), playing location (rural vs urban, streets vs parks vs at home vs other places), playing frequency (days/week, hours/day*), playing preference (alone vs with others vs none) and habit of playing (alone vs with others vs both equally), main playing motivation*.  features available at the time of data collection (i.e., raids, Community Days, raid events, field research events, PvP, trading): use (yes vs no vs don’t know what it is), frequency, solitude or social use.  friendship: number of people in the game’s friends list*, how many friends participants interact with daily in-game*and in person*, whether the participant started to play with their friends (yes vs no vs my friends don’t play), whether they met most of their current friends due to PoGo (yes vs no), how many players (whether in friends list or not) participants interact with daily*, whether participants found their partner through the game (yes vs no), and how participants spent their leisure time before playing PoGo*. |
| **Direct items** | Pokemon Go changed my life...’ 1 = for the worse – 7 = for the better, ‘I'm spending less time doing these other things since I started playing Pokemon Go’), ‘I'm spending more time outside since I started playing Pokemon Go’, ‘I'm spending more time with other people since I started playing Pokemon Go’, ‘I don't neglect important things (school, work, etc) because of Pokemon Go’), ‘I'm walking more since I started playing Pokemon Go’ |
| **Life satisfaction** | ‘I am satisfied with my life’, ‘In most ways my life is close to ideal’, ‘The conditions of my life are excellent’, ‘So far, I have gotten the important things I want in life’, ‘If I could live my life over, I would change almost nothing’, ‘I feel lonely’, ‘I am generally happy’, ‘I am satisfied with how I spend my free time’. |
| **Social functioning** | ‘I feel like I belong to a community’, ‘I spend a lot of time with my friends’, ‘I often interact with strangers’, ‘I am satisfied with my social life’, ‘I have a lot of positive social interactions’, ‘I meet up with friends on a daily basis’, ‘I am a social person/like to socialise’, ‘I have good communication skills’, ‘I find it easy to make conversation’, ‘I am satisfied with the number of friends I have’, ‘I often make new friends’, ‘I find it easy to make new friends’, ‘I find it easy to interact with strangers, with other players and non-players’ |
| **Clinical questionnaires** | 28 items Autism-Quotient (AQ; Baron-Cohen et al., 2001),  21 items Beck’s Depression Inventory (BDI; Beck et al., 1996),  20 items from the trait version of the State-Trait Anxiety Inventory (STAI; Spielberger et al., 1983) |

# **Data Preparation and Statistical Analyses**

The 7-point Likert-type items were re-coded so that 7 represented the positive end of the scale and 1 the negative end of the scale for all items. The responses to survey questions with an open answer format were categorised. That is, two variables were created from the question about how participants spent their leisure time before playing PoGo, one categorising into social vs solitude activities and one categorising into indoor vs outdoor activities. If it was not possible to categorise an answer, it was treated as a missing value and the person was not included in this particular analysis.

# **Factor Analysis on Life Satisfaction and Social Functioning**

An exploratory factor analysis was conducted on the re-coded items assessing participants’ life satisfaction and social functioning, once for the items relating to the time before and once for the time since playing PoGo. These analyses aimed at checking whether the self-formulated items would fall into the categories of life satisfaction and social functioning to be able to conduct the hypotheses testing with these factors. The items’ inter-correlations were inspected. All items correlated significantly with each other and were < .90. The Kaiser-Meyer-Olkin criterion was .935 indicating that the sample size was sufficiently large to conduct a factor analysis. The Bartlett’s test of Sphericity was significant, *χ*^2^ = 6929.09 and *χ*^2^ = 7541.68, *p*’s < .001, indicating that there were relations between the items. Factors were considered with an Eigenvalue > 1 according to the Kaiser’s criterion. A 3-factor structure was found in both pre-game and since playing analyses explaining 68% and 69% of the variance, respectively. The three factors correlated with each other exceeding the recommended minimum threshold for oblique rotation of *r* = .32 (Tabachnick et al., 2007) and, thus, oblique rotation was applied. Only items that loaded with a minimum of .40 on a factor were considered. The item ‘Satisfaction with social life’ loaded on different factors in the two analyses (before vs since playing the game) and was thus excluded. The item ‘Satisfaction with number of friends’ did not load high enough on any factor in the analysis since playing the game (i.e., < .40) and was thus excluded. These two items were excluded from their respective factors both before and since playing the game. No other items were omitted. Table S2-II presents the remaining items and their loadings on the factors. The items taken from the life satisfaction scale formed their own factor and this factor also included the added self-formulated items. The factor was thus called ‘Life satisfaction’. The factor of social functioning was split into 2 factors, ‘Sociality’ and ‘Social ability’.

**Table S2-II**

*Item Loadings on Respective Factors After Rotation*

|  | **Factors** | | |
| --- | --- | --- | --- |
| **Item** | **Factor A**  **‘Life satisfaction’** | **Factor B**  **‘Social ability’** | **Factor C**  **‘Sociality’** |
| 1 | I was/am satisfied with my life  .79/.92 | I was/am a social person/I liked/like to socialise  .53/.69 | I felt/feel like I belong to a community  .72/.63 |
| 2 | In most ways, my life was/is close to ideal  .86/.93 | I had/have good communication skills  .76/.89 | I had/have a lot of positive social interactions  .70/.56 |
| 3 | I felt/feel lonely  .43/.43 | I found/find it easy to make conversation  .83/.92 | I spent/spend a lot of time with my friends  .88/.84 |
| 4 | The conditions of my life were/are excellent  .87/.91 | I often made/make new friends  .65/.73 | I met/meet up with friends on a daily basis  .85/.87 |
| 5 | I had/have gotten the important things I want in life  .84/.90 | I often interacted/interact with strangers  .86/.79 |  |
| 6 | If I could live my life over, I would have changed/change almost nothing  .72/.78 | I found/find it easy to make new friends  .79/.92 |  |
| 7 | I was/am generally happy  .74/.77 | I found/find it easy to interact with strangers  .93/.87 |  |
| 8 | I was/am satisfied with how I spend my free time  .52/.62 |  |  |

*Note*. The item loading presented before the slash refers to the time period before playing PoGo. The item loading presented after the slash refers to the time period since playing Pokmeon Go.

# **References**

Baron-Cohen, S., Wheelwright, S., Skinner, R., Martin, J., & Clubley, E. (2001). The Autism-Spectrum Quotient (AQ): Evidence from Asperger Syndrome/High-Functioning Autism, Males and Females, Scientists and Mathematicians. *Journal of Autism and Developmental Disorders*, *31*(1), 5–17. https://doi.org/10.1023/A:1005653411471

Beck, A. T., Steer, R. A., & Brown, G. (1996). *Beck Depression Inventory–II - PsycNET* https://doi.org/10.1037/t00742-000

Spielberger, C. D., Gorsuch, R. L., Lushene, R., Vagg, P. R., & Jacobs, G. A. (1983). *Manual for the State-Trait Anxiety Inventory*.

Tabachnick, B. G., Fidell, L. S., & Ullman, J. B. (2007). *Using multivariate statistics* (Vol. 7). Boston, MA: Pearson.

1. * open response format [↑](#footnote-ref-1)
