# Supplementary material for: Playing Pokemon Go: Increased Life Satisfaction Through More (Positive) Social Interactions
Source: Front Sports Act Living. 2022 Jul 8;4:903848. doi: 10.3389/fspor.2022.903848 (PMC9304577; doi:10.3389/fspor.2022.903848)
Supplement: Supplementary file 3 [file Data_Sheet_3.docx]

# **Playing Pokemon Go: Increased Life Satisfaction Through More (Positive) Social Interactions**

**Tanja S. H. Wingenbach & Yossi Zana**

# **Supplementary Materials III: Results**

# **Effects of PoGo on Players’ Social Functioning and Life Satisfaction (Hypothesis 1)**

Paired samples *t*-tests (*N* = 434) were conducted on the three identified factors to test whether participants’ life satisfaction, sociality, and social ability were positively impacted by playing PoGo by comparing the ratings since playing PoGo to the period before playing the game. Participants reported significantly more *life satisfaction* for the period since playing the game (*M* = 4.96, *SD* = 1.29) compared to the pre-game period (*M* = 4.43, *SD* 1.43), 95% *CI* [0.44, 0.63], *t*(433) = 11.10, *p* < .001 (1-tailed), Cohen’s *d* = .54; Figure 1 in manuscript. Participants further reported significantly more *social ability* for the period since playing the game (*M* = 4.80, *SD* = 1.46) compared to the pre-game period (*M* = 4.05, *SD* = 1.62), 95% *CI* [0.64, 0.86], *t*(433) = 13.06, p < .001 (1-tailed), Cohen’s *d* = .65, and significantly more *sociality* for the period since playing the game (*M* = 5.04, *SD* = 1.33) compared to the pre-game period (*M* = 4.08, *SD* = 1.60), 95% *CI* [0.82, 1.11], *t*(433) = 12.74, *p* < .001 (1-tailed), Cohen’s *d* = .61; Figure 1 in manuscript.

In addition, one sample *t*-tests (1-tailed) were conducted to test whether the ratings significantly exceeded the midpoint of the scale (4) indicating positive evaluations of social functioning and life satisfaction. The means of the three factors of the two time periods were tested against the mid-point of the scale (4). For the period *before playing the game*, life satisfaction was significantly higher than the midpoint, 95% *CI* [0.29, 0.56], *t*(433) = 6.21, *p* < .001 (1-tailed), Cohen’s *d* = .30, but social ability and sociality were both not significantly different from the midpoint, 95% *CI* [-0.10, 0.20], *p* = .254 (1-tailed), Cohen’s *d* = .03, and 95% *CI* [-0.07, 0.23], *p* = .159 (1-tailed), Cohen’s *d* = .05, respectively (Figure 1 in manuscript). Life satisfaction was again significantly higher than the midpoint for the period *since playing the game*, 95% *CI* [0.84, 1.08], *t*(433) = 15.48, *p* < .001 (1-tailed), Cohen’s *d* = .74 (Figure 1 in manuscript). Social ability was also significantly higher for the period since playing the game, 95% *CI* [0.66, 0.94], *t*(433) = 11.42, *p* < .001 (1-tailed), Cohen’s *d* = .55, akin to sociality, 95% *CI* [0.92, 1.17], *t*(433) = 16.27, *p* < .001 (1-tailed), Cohen’s *d* = .79 (Figure 1 in manuscript).

With the results showing perceived benefits from playing PoGo on social functioning and life satisfaction and a considerable number of female participants, further analyses were conducted to examine whether the found benefits apply to both males and females. Since PoGo is available in many countries of the world, the study was launched internationally and participants were located in various parts of the world. Additional analyses were conducted to investigate whether the found benefits in social functioning and life satisfaction apply cross-culturally.

# **Additional Analyses: PoGo and Sex Differences**

Based on independent samples *t*-tests, males reported significantly higher *life satisfaction* prior to playing PoGo (*M* = 4.57, *SD* = 1.43) than females (*M* = 4.19, *SD* = 1.42), 95% *CI* [0.09, 0.67], *t*(427) = 2.61, *p* = .009 (2-tailed), Cohen’s *d* = 0.27. The difference between males (*M* = 5.04, *SD* = 1.30) and females (*M* = 4.83, *SD* = 1.26) was not significant for the period since playing the game, 95% *CI* [-0.04, 0.47], *t*(427) = 1.63, *p* = .104 (2-tailed), Cohen’s *d* = 0.16. The results are visualised in Figure S1-III. These results suggest that females perceived a greater impact of playing PoGo than males. Indeed, females (*M* = 0.62, *SD* = 0.96) had a significantly greater change score in life satisfaction (winsorised) than males (*M* = 0.44, *SD* = 0.79), 95% *CI* [0.00, 0.36], *t*(244.44) = 1.97, *p* = .025 (1-tailed), Cohen’s *d* = 0.21.

**Figure S1-III**

*Sex Differences in Life Satisfaction, Social Ability, and Sociality for the Periods Before and Since Playing Pokemon Go*
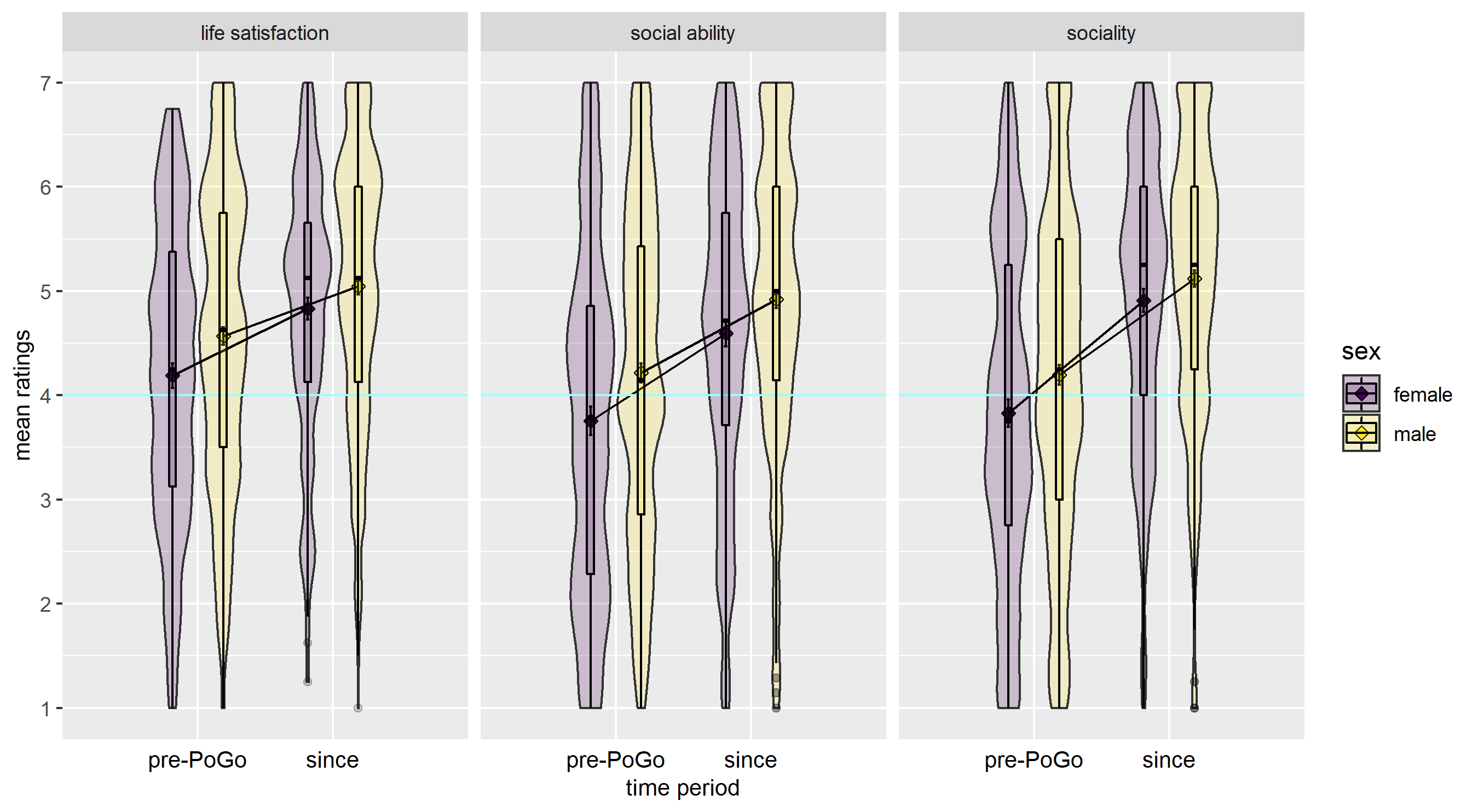


*Note*. The ratings for the time period before playing PoGo (pre-PoGO) and since playing PoGo (since) are shown. Data distributions are shown by the violin plots. The rhombus within the boxplots represents the sample mean while the black horizontal line represents the median. The blue line at the value 4 represents the middle of the rating scale, i.e., neutral rating, while all ratings above 4 represent positive and below 4 negative perceptions. Error bars represent 95% confidence intervals of the means. The diagonal lines show the increase per group.

Males (*M* = 4.22, *SD* = 1.57) reported significantly higher *social ability* than females (*M* = 3.76, *SD* = 1.65) for the pre-game period, 95% *CI* [0.14, 0.78], *t*(427) = 2.81, *p* = .005 (2-tailed), Cohen’s *d* = 0.29. However, there were no significant differences in reported social ability between males (*M* = 4.92, *SD* = 1.42) and females (*M* = 4.59, *SD* = 1.49) for the playing period, 95% *CI* [0.45, 0.72], *t*(427) = 2.22, *p* = .027 (2-tailed), Cohen’s *d* = 0.23. The results are visualised in Figure S1-III. There were no significant differences between males and females in change scores of social ability, 95% *CI* [-0.11, 0.33], *t*(243.18) = 0.99, *p* = .161 (1-tailed), Cohen’s *d* = 0.11, indicating that the perceived change in social ability was equal for both sexes.

For reported *sociality,* there were no significant differences between males (*M* = 4.19, *SD* = 1.60) and females (*M* = 3.83, *SD* = 1.59) for the pre-game period, 95% *CI* [0.04, 0.69], *t*(427) = 2.24, *p* = .026 (2-tailed), Cohen’s *d* = 0.23. The difference between males (*M* = 5.12, *SD* = 1.31) and females (*M* = 4.91, *SD* = 1.34) was also not significant for the period since playing PoGo, 95% *CI* [-0.05, 0.48], *t*(427) = 1.56, *p* = .119 (2-tailed), Cohen’s *d* = 0.16. The results are visualised in Figure S1-III.

# **Additional Analyses: PoGo and ‘Culture’**

Group allocation to the continents was done according to the reported living location as this was where they played PoGo; except for 22 participants, living location and nationality were equal. There were 112 participants from Northern America, 210 participants from Southern America, and 97 from Europe. There were 9 participants from Oceania, 2 from Africa, and 4 from Asia. Due to only few participants from Oceania, Africa, and Asia, these groups were not sufficiently large to include in the statistical analyses. Repeated measures ANOVAs were computed for each the three factors with continent as between-subject factor and time period as within-subject factor.

For *life satisfaction*, the repeated measures ANOVA results showed no significant main effect of continent, *F*(2, 416) = 0.43, *p* = .649, *η*^2^ = .00, power = .12, but a significant main effect of time period, *F*(1, 416) = 103.79, *p* < .001, *η*^2^ = .20, power = 1.00 (see Hypothesis 1). The interaction of time period and continent was not significant, *F*(2, 416) = 0.39, *p* = .676, *η*^2^ = .00, power = .11. The data are visualised in Figure S2-III.

**Figure S2-III**


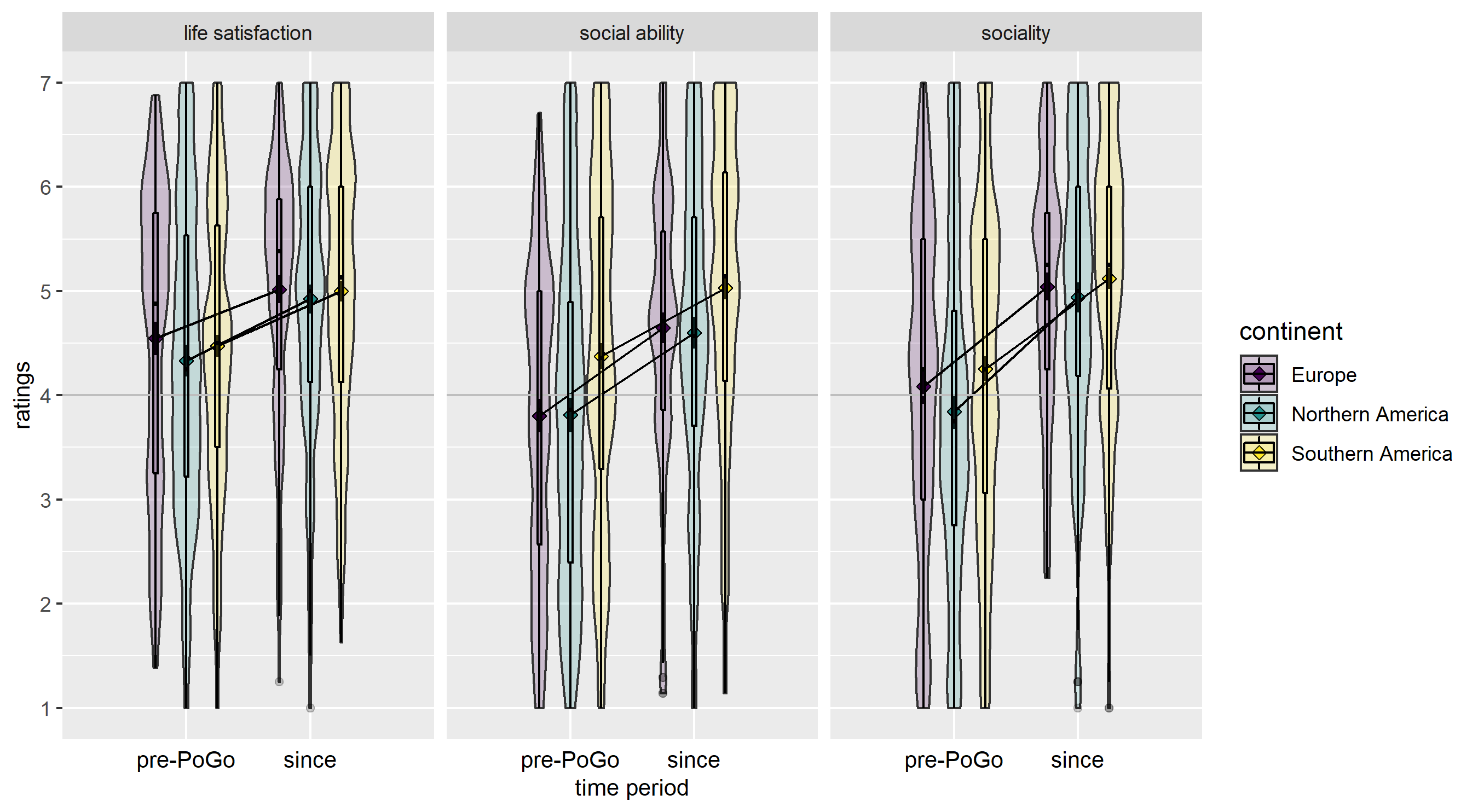
*Life Satisfaction, Social Ability, and Sociality Ratings by Continent*

*Note*. See also note Figure S1-III. The ratings on the three factors by continent and time period. There were no significant differences on life satisfaction and sociality related to continent. However, there was a main effect of continent for social ability (statistical results in text).

For *social ability*, there was a significant main effect of continent, *F*(2, 416) = 6.37, *p* = .002, *η*^2^ = .03, power = .90. Independent samples *t*-tests showed that Southern American (*M* = 4.70, *SD* = 1.44) participants reported significantly higher social ability than Northern Americans (*M* = 4.21, *SD* = 1.43), 95% *CI* [0.18, 0.82], *t*(422) = 1.58, *p* = .009 (2-tailed), Cohen’s *d* = 0.34, and Europeans (*M* = 4.23, *SD* = 1.30), 95% *CI* [0.14, 0.82], *t*(320) = 2.98, *p* = .012 (2-tailed), Cohen’s *d* = 0.34, whereas there were no significant differences between the latter two groups, 95% *CI* [-0.40, 0.36], *t*(207) = 0.03, *p* = .921 (2-tailed), Cohen’s *d* = 0.02. The main effect of time period was significant, *F*(1, 416) = 167.29, *p* < .001, *η*^2^ = .29, power = 1.00 (see Hypothesis 1), while the interaction of time period and continent was not significant, *F*(2, 416) = 1.08, *p* = .340, *η*^2^ = .01, power = .24. The data are visualised in Figure S2-III.

For *sociality*, there was no significant main effect of continent, *F*(2, 416) = 2.12, *p* = .121, *η*^2^ = .01, power = .44, but a significant main effect of time period, *F*(1, 416) = 143.45, *p* < .001, *η*^2^ = .26, power = 1.00 (see Hypothesis 1). The interaction of time period and continent was not significant, *F*(2, 416) = 0.80, *p* = .450, *η*^2^ = .00, power = .19. The data are visualised in Figure S2-III.

The majority of participants were located in Northern America, Southern America, and Europe. Life satisfaction and sociality ratings were not influenced by the continent participants were located at. However, social ability ratings were affected by continent in that Southern Americans perceived themselves as overall more socially able than Northern Americans and Europeans (independent of the time period). Whether the results extend to the rest of the world, for example Asia and Africa, has to be addressed by future research.

# **Effects of PoGo on Players’ Lifestyle**

Participants were asked to make explicit judgements on the effect of the game on them; see Supplementary Materials II, Table S2-II, ‘Direct items’. One sample *t*-tests (1-tailed) were conducted on the six items asking participants whether playing the game had an effect on their lifestyle. The testing value was 4, i.e., the neutral midpoint of the rating scale. The data showed no outliers based on 1.5x the interquartile range. Participants reported a significant positive *effect of PoGo on their life* (*M* = 5.26, *SD* = 1.18) compared to the (neutral) midpoint of the scale (4), 95% *CI* [1.15, 1.37], *t*(433) = 22.17, *p* < .001 (1-tailed), Cohen’s *d* = 1.07. Results from one sample *t*-test showed that participants reported to spend significantly more time *outdoors* because of playing PoGo compared to the (no change) midpoint of the scale (4) (*M* = 4.61, *SD* = 2.15), 95% *CI* [0.41, 0.82], *t*(433) = 5.95, *p* < .001 (1-tailed), Cohen’s *d* = 0.28. Of 434 participants, 323 (74%) reported to have had mainly leisure activities that take place inside with 18 (4%) reporting outside activities and 42 both (10%) before they played PoGo; for the remaining participants, this could not be determined based on their response. Further, 314 (72%) participants reported solitude leisure time activities, 23 (5%) reported social activities, and 27 both (6%) before they played PoGo. Again, for the remaining participants, this could not be determined. Results from one sample *t*-test showed that participants reported to *socialise* significantly more because of playing PoGo compared to the midpoint of the scale (4) (*M* = 4.29, *SD* = 1.95), 95% *CI* [0.10, 0.47], *t*(433) = 3.08, *p* = .001 (1-tailed), Cohen’s *d* = 0.15, and to be significantly more physically *active* (*M* = 5.15, *SD* = 2.19), 95% *CI* [0.94, 1.35], *t*(433) = 10.88, *p* < .001 (1-tailed), Cohen’s *d* = 0.53.

Participants did not report to a significant degree that *PoGo replaced their previous hobbies* (*M* = 3.85, *SD* = 1.89) compared to the midpoint of the scale (4), 95% *CI* [-0.33, 0.03], *t*(433) = -1.63, *p* = .104 (1-tailed), Cohen’s *d* = -0.08. Participants did not report significant *interference with necessities in their life* due to playing the game compared to the midpoint of the scale (4) (*M* = 4.18, *SD* = 2.35), 95% *CI* [-0.04, 0.40], *t*(433) = 1.59, *p* = .112 (1-tailed), Cohen’s *d* = 0.08.

Players judged the effects of playing PoGo on their lifestyle as entirely positive. That is, players perceived the game to have an overall positive effect on them, positively affect their social interactions and increase their physical activity and time spent outside, while players did not perceive the game to interfere with important things in their life or to replace pre-existing hobbies. These results are in line with a previous study on explicit judgements where 57% of PoGo players reported to be more motivated to go outside, 55% reported that playing PoGo made them happier, and 64% reported to play the game together with others (Ruiz-Ariza et al., 2018). Such findings show that participants perceived a direct effect of the game on their life, which they further perceived as beneficial. While participants in the Ruiz-Ariza et al. (2018) study were randomly allocated to the playing PoGo group and 12-15 years of age, the current study extends the findings to adult existing players, 18-69 years of age. A possible mechanism behind participants’ increased content with their social interactions is the finding from the current study that many participants (32%) stated to have made most of their friends and some to have found their romantic partner by means of playing PoGo (*n* = 27). These reports align with the finding of friendship maintenance and relationship initiation as a playing motivations for PoGo (Yang & Liu, 2017). Thus, PoGo can be seen as a tool to introduce more healthy leisure time habits and facilitate the forming of relationships both romantic partnerships and friendships.

# **Playing Motivations and Change in Social Functioning/Life Satisfaction**

Different playing motivations might differentially impact players. A study comparing the effects of three playing motivations for PoGo, i.e. immersion, achievement, and social interaction, found differential effects on perceived health outcomes- physical, mental, and social (Koivisto et al., 2019). That is, immersion and achievement were positively linked with mental and physical health while social interaction was strongly associated with all three health aspects. It can be assumed that individual playing motivations would differentially affect the change in life satisfaction and social functioning from before to since playing PoGo, e.g., social playing motivations increase perceived social functioning. This was explored. Playing motivation was assessed with an open response format. The responses to the question asking for playing reasons with an open response format were grouped to form broader categories: fun, exercise, filler activity, exploration, gaming addiction, purpose, distraction, socialising.

The category *fun* includes responses of enjoyment in general and to specific aspects of the game (e.g., shiny hunting) and was the most popular response (*n* = 308), followed by *socialising* (*n* = 85), and *physical activity* (*n* = 64). Physical activity as a category refers to activities such as walking and running. Other playing reasons reported were using the game as *filler activity* (*n* = 23), e.g., during the commute to work, *distraction* (*n* = 24) from negative things happening in life, *purpose* (*n* = 20), i.e., as a motivation to get out of bed or out of the house, *relaxation* (*n* = 7), *gaming addiction* (*n* = 5), and *mental exercise* (*n* = 2), e.g., learning the names of all the Pokemon.

Change scores were created for the three factors, subtracting the since playing value from the pre-game value to retrieve variables representing the effect of the game on participants. The change scores were visually inspected for outliers using boxplots grouped by playing motivation. Variables were winsorised based on the 25^th^ and 75^th^ percentiles of the Tukey's Hinges to account for outliers. Since it is possible that the effect from playing the game on individuals is dependent on their playing reason, one sample *t*-tests (1-tailed) were conducted for the playing motivations with a minimum *n* of 20 using the change scores of the 3 factors against a test value of 0 (i.e., no change). Even though participants were asked to state their main reason for playing, they often reported several playing motivations (without ranking). Thus, some participants were included more than once in the analyses and the results should be interpreted with care.

Table S1-III shows the change scores for life satisfaction, social ability and sociality factors per identified playing reason. Results from the one sample *t*-tests on the change scores for the playing motivations are as follows: Participants who reported *fun* as playing reason reported a significant change in life satisfaction, *t*(307) = 10.42, *p* < .001 (1-tailed), Cohen’s *d* = 0.59, social ability, *t*(307) = 12.68, *p* < .001 (1-tailed), Cohen’s *d* = 0.73, and sociality, *t*(307) = 10.99, *p* < .001 (1-tailed), Cohen’s *d* = 0.63. Participants who reported *socialising* as playing reason reported a significant change in life satisfaction, *t*(84) = 5.70, *p* < .001 (1-tailed), Cohen’s *d* = 0.62, social ability, *t*(84) = 8.78, *p* < .001 (1-tailed), Cohen’s *d* = 0.95, and sociality, *t*(84) = 9.65, *p* < .001 (1-tailed), Cohen’s *d* = 1.05. Participants who reported *physical activity* as playing reason reported a significant change in life satisfaction, *t*(63) = 6.99, *p* < .001 (1-tailed), Cohen’s *d* = 0.88, social ability, *t*(63) = 5.97, *p* < .001 (1-tailed), Cohen’s *d* = 0.74, and sociality, *t*(63) = 5.78, *p* < .001 (1-tailed), Cohen’s *d* = 0.72. Participants who reported *distraction* as playing reason reported a significant change in life satisfaction, *t*(23) = 3.18, *p* = .004 (1-tailed), Cohen’s *d* = 0.65, social ability, *t*(23) = 2.71, *p* = .013(1-tailed), Cohen’s *d* = 0.55, and sociality, *t*(23) = 3.27, *p* = .003 (1-tailed), Cohen’s *d* = 0.67. Participants who reported *purpose* as playing reason reported a significant change in life satisfaction, *t*(19) = 3.66, *p* = .002 (1-tailed), Cohen’s *d* = 0.82, social ability, *t*(19) = 3.37, *p* = .003 (1-tailed), Cohen’s *d* = 0.76, and sociality, *t*(19) = 4.10, *p* = .001 (1-tailed), Cohen’s *d* = 0.92. However, participants who reported *filler activity* as playing reason did not report a significant change in life satisfaction, *t*(22) = 0.27, *p* = .787 (1-tailed), Cohen’s *d* = 0.06, social ability, *t*(22) = 1.68, *p* = .106 (1-tailed), Cohen’s *d* = 0.35, or sociality, *t*(22) = 1.09, *p* = .289 (1-tailed), Cohen’s *d* = 0.23.

**Table S1-III**

*Change Scores for the Three Factors per Playing Reason*

| **Playing reason** | **Δ life satisfaction**  ***M* (*SD*)**  **95% CI** | **Δ social ability**  ***M* (*SD*)**  **95% CI** | **Δ sociality**  ***M* (*SD*)**  **95% CI** |
| --- | --- | --- | --- |
| fun  (*n* = 308) | 0.48 (0.81)***  0.39-0.57 | 0.69 (0.95)***  0.58-0.79 | 1.45 (1.51)***  0.74-1.07 |
| **socialising**  (*n* = 85) | 0.61 (0.98)***  0.40-0.82 | **1.16 (1.22)*****  **0.90-1.42** | **1.60 (1.53)*****  **1.27-1.93** |
| physical activity  (*n* = 64) | 0.86 (0.98)***  0.61-1.10 | 0.92 (1.23)***  0.61-1.22 | 1.25 (1.73)***  0.81-1.68 |
| **distraction**  (*n* = 24) | **0.96 (1.48)***  **0.34-1.59** | 1.01 (1.82)*  0.24-1.77 | 1.50 (2.25)*  0.55-2.45 |
| filler activity  (*n* = 23) | 0.04 (0.66)  0.25-0.32 | 0.22 (0.63)  -0.05-0.49 | 0.16 (0.71)  -0.15-0.47 |
| purpose  (*n* = 20) | 0.69 (0.84)**  0.29-1.08 | 0.69 (0.91)*  0.26-1.11 | 1.24 (1.35)**  0.61-1.87 |

*Note*. Change scores of the three factors were tested against 0 (= no change). 95% CI = of the difference. Distraction as playing motivation had the greatest change effect on life satisfaction whereas socialising as playing motivation had the greatest effect of change on social ability as well as sociality (marked in boldface).

****p* < .001, ***p* < .01, **p* < .05 (1-tailed).

Individual playing motivations differentially affected the change in life satisfaction and social functioning from before to since playing PoGo. Players who reported to be motivated by fun, those who were motivated by the opportunity for socialising, those who used PoGo as distraction, those who perceived the game to provide them with purpose, and those who were motivated by the required physical activity reported significant perceived benefits on social functioning and life satisfaction. Only players who used the game as a filler activity perceived no significant benefits on social functioning and life satisfaction since playing PoGo. The most prominent changes were reported by players who played PoGo for social reasons or for distraction. Players who played for the social aspects of the game perceived a strong increase on their social functioning. Players who used the game for distraction saw benefits for social functioning and life satisfaction. A study on adult Japanese workers who were actively playing PoGo over a year found significantly reduced levels of psychological distress (Watanabe et al., 2017). It is conceivable that distraction decreases distress and thereby increases perceived life satisfaction. As such, playing PoGo could be used to modulate life satisfaction. In general, the changes participants reported since playing the game in the current study seem to align with the reasons for their continued gameplay, or in other words, players seem to fulfil some of their needs through playing the game.

# **Model Predicting Changes in Life Satisfaction (Hypothesis 2)**

Based on the known importance of social interactions for humans and their life satisfaction, the following model of effect was investigated: The perceived change in social ability affects the perceived change in life satisfaction and is mediated by the perceived change in sociality, which in turn is modulated by the quantity of daily interactions with other PoGo players (see Supplementary Materials II, Figure S1-II).

To clarify whether the interactions with others have to be in person or can be in game and whether interaction partners should be from a player’s friends list, linear regression analyses were conducted for each of the change scores of the three factors identified with the factor analyses and participants’ reports on social interaction extent, in person (‘With how many PoGo players do you interact on a daily basis in person?’, ‘With how many people from your friends list do you interact in person on a daily basis?’) and in game (‘With how many people from your friends list do you interact daily in game?’) as predictors. The predictors were entered stepwise. All variables were winsorised based on the 25^th^ and 75^th^ percentiles of the Tukey's Hinges to account for outliers. The residuals were normally distributed.

Regression results showed that the number of face-to-face interactions with other PoGo players on a daily basis was identified as significant predictor of the *life satisfaction* change score, *F*(1, 432) = 14.50, *p* < .001, *r*^2^ = .03. The resulting equation was: change score in life satisfaction = 0.35 + (0.03 * players interaction/day). The number of face-to-face interactions with other PoGo players on a daily basis was also identified as significant predictor of the social ability change score, *F*(1, 432) = 27.04, *p* < .001, *r*^2^ = .06. The resulting equation was: change score in *social ability* = 0.48 + (0.04 * players interaction/day). The number of face-to-face interactions with other PoGo players on a daily basis was further identified as significant predictor of the *sociality* change score, *F*(1, 432) = 33.46, *p* < .001, *r*^2^ = .07. The resulting equation was: change score in sociality = 0.56 + (0.06 * players interaction/day). (The face-to-face interactions with players in one’s friends list and in-game interactions with players were not found to be significant predictors). Results showed that the extent of face-to-face interactions matters more for the changes score than in-game interactions and that face-to-face interaction partners do not have to be from a player’s in-game friend’s list. Thus, the number of daily face-to-face interactions with other players was included in the moderated mediation model to predict the change in life satisfaction.

Model number 14 of the Hayes Process Macro for SPSS was used (Hayes, 2015). Table 1 in manuscript shows all effects. The direct effect of the change in social ability on the change in sociality was significant. The change in social ability and the change in sociality both significantly predicted the change in life satisfaction whereas the quantity of daily face-to-face player interactions was not significantly associated with the change in life satisfaction. There was no significant interaction between the change in social ability and the change in sociality (X*M), *F*(1, 428) = 1.24, *p* = .266. The unconditional interaction between the change in sociality and the quantity of daily face-to-face player interactions was significant (M*W), Δ*r*^2^ = .01, *F*(1, 429) = 6.58, *p* = .011, 95% *CI* [0.001, 0.01], indicating moderated mediation.

Based on 5,000 bootstrap samples with a 95% *CI* at the 16^th^ and 84^th^ percentiles, the moderated mediation was significant. Bootstrap results showed significant effects at -1 *SD* (player interaction = 1, *b* = 0.16, *SE* = 0.05, 95% *CI* [0.07, 0.26]), *M* (player interaction = 4.5, *b* = 0.19, *SE* = 0.05, 95% *CI* [0.10, 0.28]), and +1 *SD* of the moderator (player interaction = 10, *b* = 0.22, *SE* = 0.05, 95% *CI* [0.13, 0.31]) on the indirect effect; Figure S3-III. The direct effect model of the change in social ability on the change in sociality explained 53% of the variance; the moderated mediation model predicting the change in life satisfaction explained 35% of the variance.

**Figure S3-III**

*Conditional Effects*


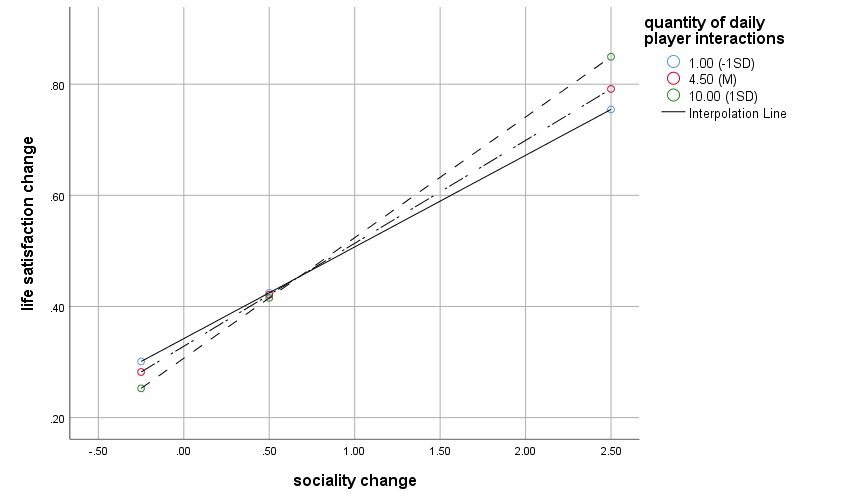


*Note*. Conditional effects of the focal predictor at values of the moderator (-1 *SD*, *M*, 1 *SD*), M*W on Y. The three slopes are significant, *p*’s < .001. With increasing change in sociality life satisfaction increases and the more daily face-to-face player interactions players have, the greater the effect.

# **PoGo Social Play Habits and Preferences (Hypothesis 3)**

It was investigated whether people who prefer to and mainly do play alone benefit from playing PoGo as do players who prefer and mainly engage in group play. It was hypothesised that a more social gameplay leads to greater increases in social functioning and life satisfaction than solitude game play. Participants were grouped based on their stated preferences of social play (alone, in a group, both equally) in combination with their stated playing habit (alone/group/both) to investigate whether these groups differed on the three factors for the time periods of before and since playing the game, and whether playing the game affected the groups differentially. Whereas this should lead to nine groups, the combinations of no preference and group play occurred in three participants, preference of playing alone and playing in groups did not occur, and preference of playing alone and playing alone and in groups to equal parts occurred in 14 participants. These group sizes were too small to be considered in the statistical analysis and were thus omitted. With the remaining groups, a 2 (time period) x 6 (group) repeated measures ANOVA was conducted separately for life satisfaction, sociality and social ability. Significant main effects were qualified with *t*-tests (1-tailed). The majority of participants stated to prefer group play and play alone and in groups to equal parts (*n* = 107), followed by no preference and playing alone and in groups to equal parts (*n* = 90), no preference and playing alone (*n* = 61), preferring groups and mainly playing alone (*n* = 60), preferring to play alone mainly playing alone (*n* = 60), and lastly preferring group play and mainly playing in groups (*n* = 38).

For *life satisfaction*, there was a significant main effect of time period, *F*(1, 410) = 110.09, *p* < .001, *η*^2^ = .21, power = 1.00 (see also Hypothesis 1) and no significant main effect of group, *F*(5, 410) = 1.05, *p* = .387, *η*^2^ = .01, power = .38. There was a significant interaction of time period and group, *F*(5, 410) = 2.99, *p* = .012, *η*^2^ = .04, power = .86. MANOVA was conducted to qualify the interaction with group as between-subject factor and the life satisfaction ratings from the two time periods as DVs. Results showed no significant main effect of group, neither for the period before playing PoGo, *F*(5, 410) = 1.21, *p* = .304, *η*^2^ = .02, power = .43, nor for the period since playing the game, *F*(5, 410) = 1.43, *p* = .213, *η*^2^ = .02, power = .50. Paired samples *t*-tests were thus conducted to qualify the interaction and the time periods were compared for each group to examine whether or not life satisfaction significantly increased in all groups. Results showed that the increase in perceived life satisfaction from the pre-game period to since playing PoGo was significant in all groups (Table S2-III).

**Table S2-III**

*Life Satisfaction Before and Since playing PoGo by Preference and Playing Habit*

| **Preference, habit** | **Pre-PoGo**  ***M (SD)*** | **Since**  ***M (SD)*** | **95% CI of the difference** |  |
| --- | --- | --- | --- | --- |
| alone, alone  *n* = 60 | 4.51 (1.39) | 4.75 (1.32) | -0.45, -0.03 | *t*(59) = -2.33, *p* = .012, *d* = 0.30 |
| group, alone  *n* = 60 | 4.09 (1.34)^#^ | 4.82 (1.42) | -0.97, -0.49 | *t*(59) = -6.03, *p* < .001, *d* = 0.82 |
| group, group  *n* = 38 | 4.60 (1.59) | 5.35 (1.24) | -1.19, -0.31 | *t*(37) = -3.43, *p* = .001, *d* = 0.56 |
| group, both  *n* = 107 | 4.48 (1.33) | 5.09 (1.14) | -0.81, -0.40 | *t*(106) = -5.74, *p* < .001, *d* = 0.56 |
| none, alone  *n* = 61 | 4.65 (1.49) | 4.93 (1.46) | -0.46, -0.08 | *t*(60) = -2.89, *p* = .003, *d* = 0.38 |
| none, both  *n* = 90 | 4.36 (1.40) | 4.95 (1.15) | -0.78, -0.40 | *t*(89) = -6.04, *p* < .001, *d* = 0.64 |

*Note*. ‘Preference’ refers to whether players prefer to play alone, with others or have no preference and ‘habit’ refers to whether players actually play with others, alone, or both to equal parts.

^#^This mean was not significantly different from the neutral midpoint of the scale, *t*(59) = 0.45, *p* = .326; all other means were significantly above the midpoint *p*’s < .013. *P*-values are based on 1-tailed testing.

The obtained group means for the two time periods suggest that the change in life satisfaction was different for the groups and thus, the change scores in life satisfaction were compared between the groups using ANOVA. (This variable was winsorised to make the outliers less extreme). Results showed a significant main effect of group, *F*(5, 410) = 3.24, *p* = .007, *η*^2^ = .04, power = .89. The change score was highest for players who prefer to play in groups but actually mainly play alone (*M* = 0.73, *SD* = 0.93), followed by players who prefer to play in groups but actually play alone and in groups to equal parts (*M* = 0.60, *SD* = 0.96), those with no preference and actually play alone and in groups to equal parts (*M* = 0.57, *SD* = 0.87), those who prefer and mainly do play in groups (*M* = 0.55, *SD* = 0.81), those who have no preference but mainly play alone (*M* = 0.29, *SD* = 0.66), and those who prefer to and mainly do play alone reported the lowest change in life satisfaction (*M* = 0.24, *SD* = 0.63). Significant group differences are displayed in Figure S4-III.

**Figure S4-III**

*Change in Life Satisfaction*


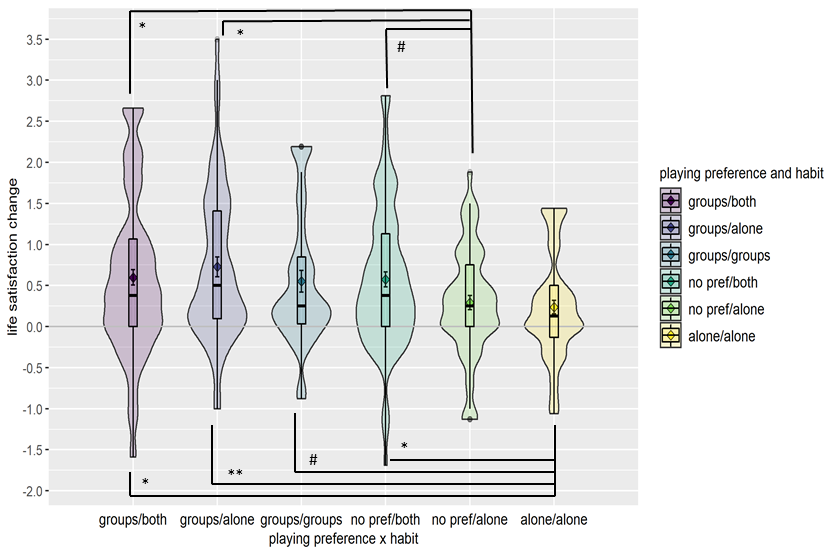


*Note*. Change in life satisfaction from before to since playing PoGo by playing preference and habit. **p < .01, *p < .05, ^#^p < .074 (1-tailed).

For *social ability*, there was a significant main effect of time period, *F*(1, 410) = 148.01, *p* < .001, *η*^2^ = .27, power = 1.00 (see also Hypothesis 1) and a significant main effect of group, *F*(5, 410) = 2.47, *p* = .032, *η*^2^ = .03, power = .78. Given the significant interaction of time period and group, *F*(5, 410) = 3.23, *p* = .007, *η*^2^ = .04, power = .89, and its disordinal nature, the interaction, not the main effects, was followed up. The data underlying the interaction are visualised in Figure S5-III. MANOVA was conducted with group as between-subject factor and the social ability ratings from the two time periods as DVs. Results showed no significant main effect of group for the period before playing PoGo, *F*(5, 410) = 0.83, *p* = .533, *η*^2^ = .01, power = .30, but for the period since playing the game, *F*(5, 410) = 4.89, *p* < .001, *η*^2^ = .06, power = .98. Thus, the groups were compared to each other for the period since playing the game on their social ability ratings using independent samples *t*-tests (Table S3-III).

**Figure S5-III**

*Social Ability Ratings by Time Period and Group***
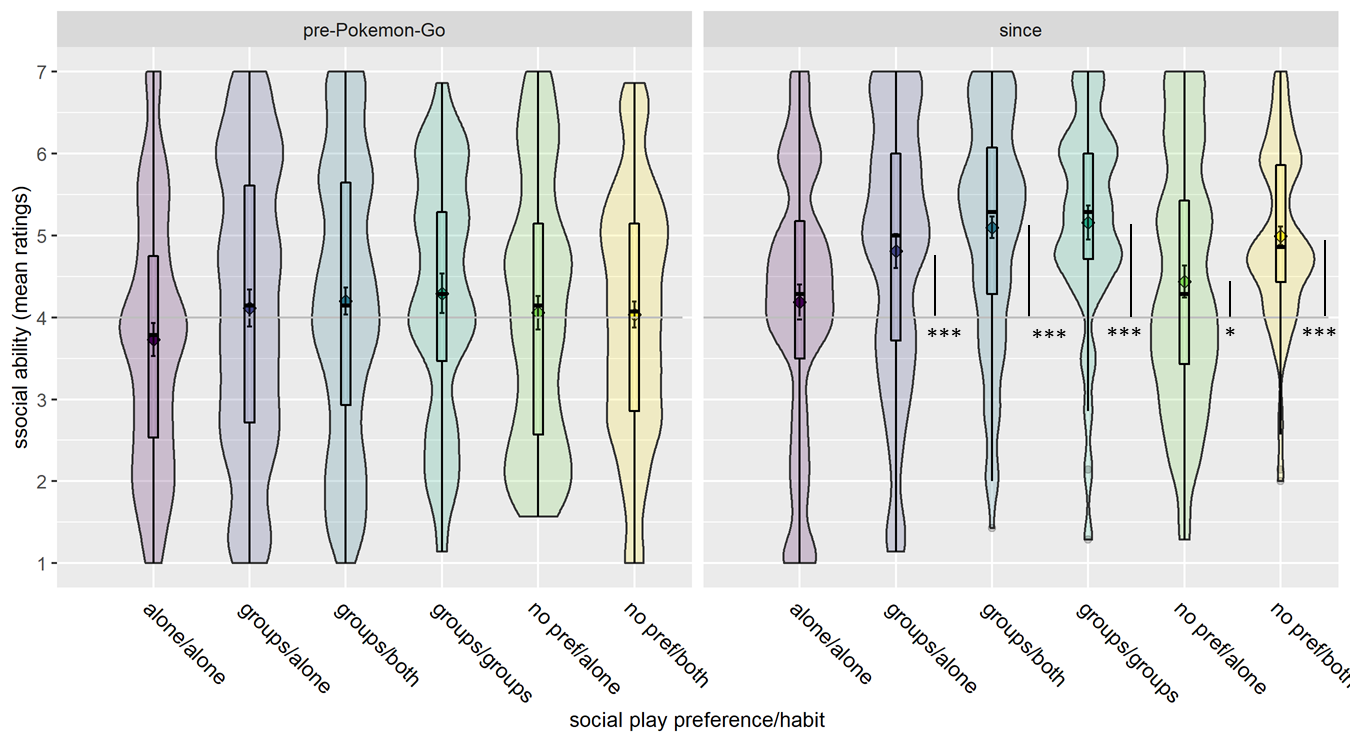
***Note*. There were no significant group differences for the time period of before playing PoGo; the group differences statistics for the time period since playing PoGo are presented in Table S3-III. The figure also displays whether the ratings were significantly above the midpoint of the scale per group, where there were only significant findings for the time period since playing the game.

****p* < .001, **p* = .015 (1-tailed).

**Table S3-III**

*Social Ability Group Comparisons Since Playing PoGo by Preference and Playing Habit*

| ***M (SD)*** | | ***M (SD)*** | **95% *CI* of the difference** |  |  |  |
| --- | --- | --- | --- | --- | --- | --- |
| **alone, alone**  *n* = 60  4.19 (1.65) | **group, alone**  *n* = 60  4.81 (1.61) | | -1.21, -0.04 | *t*(118) = -2.10, *p* = .038,  *d* = 0.38 | | |
| **alone, alone**  *n* = 60  4.19 (1.65) | **group, group**  *n* = 38  5.16 (1.29) | | -1.60, -0.34 | *t*(96) = -3.08, *p* = .006,  *d* = 0.64 | | |
| **alone, alone**  *n* = 60  4.19 (1.65) | **group, both**  *n* = 107  5.10 (1.36) | | -1.38, -0.44 | *t*(165) = -3.85, *p* < .001,  *d* = 0.62 | | |
| alone, alone  *n* = 60  4.19 (1.65) | no pref, alone  *n* = 61  4.44 (1.53) | | -0.82, 0.32 | *t*(119) = -0.87, *p* = .195,  *d* = 0.16 | | |
| **alone, alone**  *n* = 60  4.19 (1.65) | **no pref, both**  *n* = 90  5.00 (1.05) | | -1.24, -0.37 | *t*(148) = -3.66, *p* = .006,  *d* = 0.61 | | |
| group, alone  *n* = 60  4.82 (1.61) | group, group  *n* = 38  5.16 (1.29) | | -0.96, 0.27 | *t*(96) = -1.12, *p* = .388,  *d* = 0.23 | | |
| group, alone  *n* = 60  4.82 (1.61) | group, both  *n* = 107  5.10 (1.36) | | -0.75, 0.18 | *t*(165) = -1.22, *p* = .388,  *d* = 0.19 | | |
| group, alone  *n* = 60  4.82 (1.61) | no pref, alone  *n* = 61  4.44 (1.53) | | -0.19, 0.94 | *t*(119) = 1.31, *p* = .095,  *d* = 0.24 | | |
| group, alone  *n* = 60  4.81 (1.61) | no pref, both  *n* = 90  5.00 (1.05) | | -0.65, 0.28 | *t*(92.57) = -0.78, *p* = .388,  *d* = 0.14 | | |
| group, group  *n* = 38  5.16 (1.29) | group, both  *n* = 107  5.10 (1.36) | | -0.44, 0.56 | *t*(143) = 0.23, *p* = .458,  *d* = 0.05 | | |
| **group, group**  *n* = 38  5.16 (1.29) | **no pref, alone**  *n* = 61  4.44 (1.53) | | 0.13, 1.31 | *t*(97) = 2.42, *p* = .036,  *d* = 0.50 | | |
| group, group  *n* = 38  5.16 (1.29) | no pref, both  *n* = 90  5.00 (1.05) | | -0.27, 0.59 | *t*(126) = 0.75, *p* = .458,  *d* = 0.14 | | |
| **group, both**  *n* = 107  5.10 (1.36) | **no pref, alone**  *n* = 61  4.44 (1.53) | | 0.21, 1.11 | *t*(166) = 2.90, *p* = .008,  *d* = 0.46 | | |
| ***M (SD)*** | ***M (SD)*** | | **95% *CI* of the difference** |  | | |
| group, both  *n* = 107  5.10 (1.36) | no pref, both  *n* = 90  5.00 (1.05) | | -0.24, 0.44 | *t*(193.81) = 0.60, *p* = .548,  *d* = 0.08 | | |
| **no pref, alone**  *n* = 61  4.44 (1.53) | **no pref, both**  *n* = 90  5.00 (1.05) | | -1.00, -0.11 | *t*(198.03) = -2.48, *p* = .008, *d* = 0.44 | | |

*Note*. Preference refers to whether players prefer to play alone, with others, or have no preference, and habit refers to whether players actually play with others, alone, or both to equal parts. Groups that are significantly different from each other are highlighted in boldface (per line). *P*-values are based on 1-tailed testing.

It was further examined whether the social ability ratings per group from before playing PoGo differed significantly from the period since playing the game using paired samples *t*-tests. In line with the hypothesis, results showed that the social ability ratings were significantly higher since playing PoGo than before for all groups; Table S4-III. As demonstrated in Figure S5-III, one sample *t*-tests showed social ability ratings significantly above the neutral midpoint of the rating scale (i.e. positive ratings) for the period since playing PoGo for the group who prefers to play in groups but mainly plays alone, 95% *CI* [0.40, 1.23], *t*(59) = 3.91, *p* < .001 (1-tailed), *d* = 0.51, participants who prefer and also do play in groups, 95% *CI* [0.73, 1.58], *t*(37) = 5.53, *p* < .001 (1-tailed), *d* = 0.90, participants who prefer to play in groups and play alone and in groups to equal parts, 95% *CI* [0.84, 1.36], *t*(106) = 8.37, *p* < .001 (1-tailed), *d* = 0.81, participants who have no preference and play alone and in groups to equal parts, 95% *CI* [0.77, 1.22], *t*(89) = 8.96, *p* < .001 (1-tailed), *d* = 0.95, and participants who have no preference and mainly play alone, 95% *CI* [0.05, 0.83], *t*(60) = 2.34, *p* = .015 (1-tailed), *d* = 0.29. The ratings were not significantly above the midpoint of the scale for the group who prefers to and mainly plays alone, 95% *CI* [-0.45, 1.43], *t*(59) = 0.89, *p* = .190 (1-tailed), *d* = 0.12. None of the groups’ ratings were significantly different from the midline of the rating scale for the time period before playing PoGo, *t*’s(37-106) = -1.34-1.22, *p*’s = .093-.417 (1-tailed); Figure S5-III.

**Table S4-III**

*Social Ability Before and Since Playing PoGo by Preference and Playing Habit*

| **Preference, habit** | **Pre-PoGo**  ***M (SD)*** | **Since**  ***M (SD)*** | **95% CI of the difference** |  |
| --- | --- | --- | --- | --- |
| alone, alone  *n* = 60 | 3.73 (1.55) | 4.19 (1.65) | -0.74, -0.17 | *t*(59) = -3.20, *p* = .001, *d* = 0.42 |
| group, alone  *n* = 60 | 4.11 (1.76) | 4.82 (1.61) | -1.00, -0.39 | *t*(59) = -4.58, *p* < .001, *d* = 0.60 |
| group, group  *n* = 38 | 4.29 (1.49) | 5.16 (1.29) | -1.25, -0.48 | *t*(37) = -4.55, *p* < .001, *d* = 0.74 |
| group, both  *n* = 107 | 4.20 (1.71) | 5.10 (1.36) | -1.13, -0.67 | *t*(106) = -7.69, *p* < .001, *d* = 0.74 |
| none, alone  *n* = 61 | 4.06 (1.61) | 4.44 (1.53) | -0.56, -0.20 | *t*(60) = -4.19, *p* < .001, *d* = 0.54 |
| none, both  *n* = 90 | 4.03 (1.50) | 5.00 (1.05) | -1.22, -0.71 | *t*(89) = -7.53, *p* < .001, *d* = 0.80 |

*Note*. ‘Preference’ refers to whether players prefer to play alone, with others, or have no preference and ‘habit’ refers to whether players actually play with others, alone, or both to equal parts. *P*-values are based on 1-tailed testing.

For *sociality*, there was a significant main effect of time period, *F*(1, 410) = 126.93, *p* < .001, *η*^2^ = .24, power = 1.00 (see also Hypothesis 1) and a significant main effect of group, *F*(2, 430) = 11.56, *p* < .001, *η*^2^ = .05, power = .99. There was also a significant interaction of time period and group, *F*(5, 410) = 5.24, *p* < .001, *η*^2^ = .06, power = .99 (Figure S6-III). MANOVA was conducted with group as between-subject factor and the social ability ratings from the two time periods as DVs. Results showed a significant main effect of group for the period before playing PoGo, *F*(5, 410) = 2.40, *p* = .037, *η*^2^ = .03, power = .76, and for the period since playing the game, *F*(5, 410) = 12.42, *p* < .001, *η*^2^ = .13, power = 1.00. Given the significant main effects, the groups were compared to each other for the two time points separately using independent samples *t*-tests (Table S5-III); the results are in line with the hypothesis.

**Figure S6-III**

*Sociality Ratings*

*
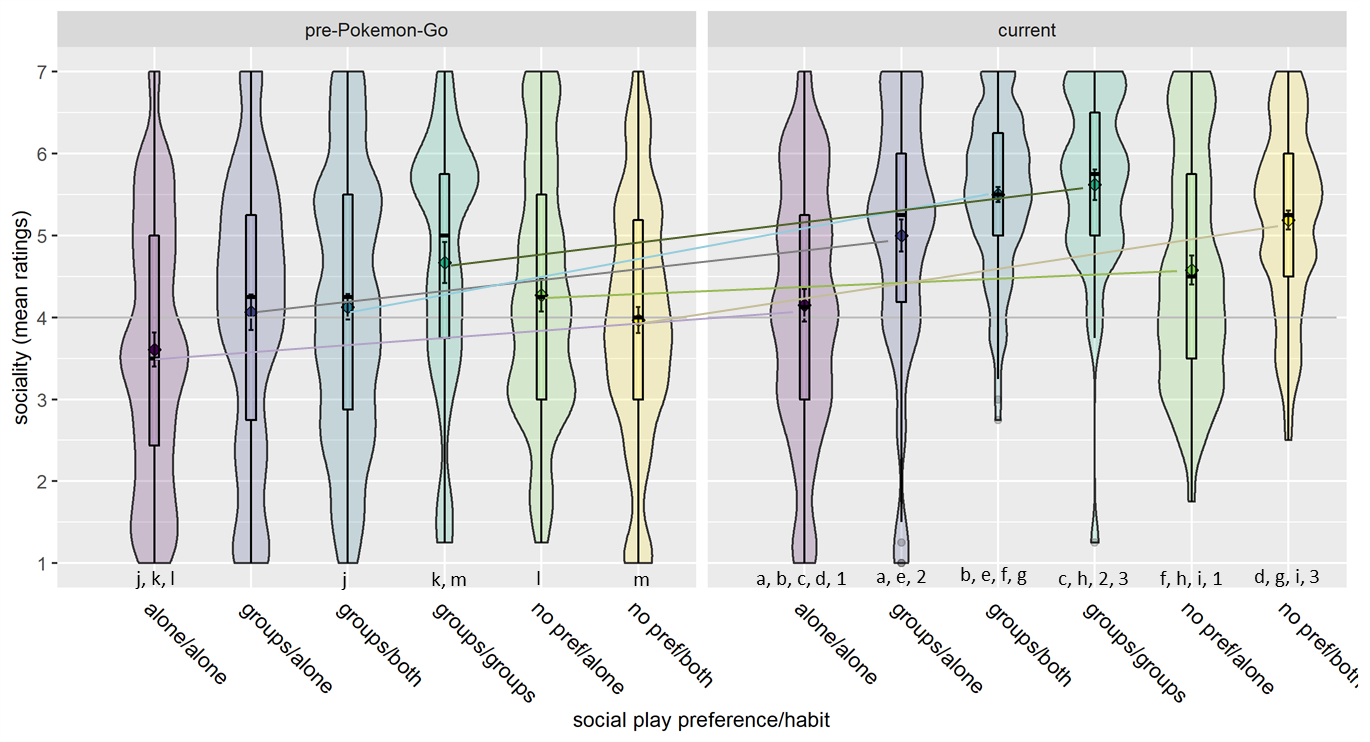
*

*Note*. Sociality ratings for the time periods before and since playing PoGo by social play preference and habit. alone/alone = players who prefer to and do play mainly alone, groups/alone = players who prefer to play in groups but mainly play alone, groups/both = players who prefer to play in groups but play in groups and alone to equal parts, no pref/alone = players who do not have a preference but mainly play alone, no pref/both = players who do not have a preference and play in groups and alone to equal parts. Letters represent significant differences between equal letter pairs; numbers represent trends towards significant differences between equal letter pairs.

|  | **alone/**  **alone**  **(*n* = 60)** | **groups/**  **alone**  **(*n* = 60)** | **groups/**  **both**  **(*n* = 107)** | **groups/**  **groups**  **(*n* = 38)** | **no pref/**  **alone**  **(*n* = 61)** | **no pref/**  **both**  **(*n* = 90)** |
| --- | --- | --- | --- | --- | --- | --- |
| **alone/**  **alone**  **(*n* = 60)** | - | 95% *CI*  [-1.40, -0.30], *t*(118) = -3.05,  *p* = .004^a^, *d* = 0.56 | 95% *CI*  [-1.79, -0.91], *t*(85.65) = -6.17,  *p* < .001^b^, *d* = 1.13 | 95% *CI*  [-2.01, -0.93], *t*(93.06) = -5.39,  *p* < .001^c^, *d* = 1.05 | 95% *CI*  [-0.96, 0.10],  *t*(119) = -1.60,  *p* = .056^1^, *d* = 0.29 | 95% *CI*  [-1.49, -0.58], *t*(98.19) = -4.52,  *p* < .001^d^, *d* = 0.81 |
| **groups/**  **alone**  **(*n* = 60)** | 95% *CI*  [-1.05, 0.14], *t*(118) = -1.51,  *p* = .134, *d* = 0.27 | - | 95% *CI*  [-0.93, -0.07], *t*(86.19) = -2.30,  *p* = .048^e^, *d* = 0.42 | 95% *CI*  [-1.19, -0.05],  *t*(96) = -2.28,  *p* = .051^2^, *d* = 0.45 | 95% *CI*  [-0.10, 0.95],  *t*(119) = 1.59,  *p* = .114, *d* = 0.29 | 95% *CI*  [-0.64, 0.27],  *t*(98.91) = -0.82,  *p* = .275, *d* = 0.15 |
| **groups/**  **both**  **(*n* = 107)** | 95% *CI*  [-1.04, -0.00], *t*(165) = -1.99,  *p* = .075^j^, *d* = 0.32 | 95% *CI*  [-0.59, 0.46],  *t*(165) = -0.25,  *p* = .720, *d* = 0.04 | - | 95% *CI*  [-0.50, 0.26],  *t*(143) = -0.62,  *p* = .269, *d* = 0.12 | 95% *CI*  [0.52, 1.32], *t*(93.05) = 4.57,  *p* < .001^f^, *d* = 0.81 | 95% *CI*  [0.02, 0.60],  *t*(195) = 2.14,  *p* = .036^g^, *d* = 0.30 |
| **groups/**  **groups**  **(*n* = 38)** | 95% *CI*  [-1.71, -0.41], *t*(96) = -3.24,  *p* = .010^k^, *d* = 0.67 | 95% *CI*  [-1.28, 0.06],  *t*(96) = -1.80,  *p* = .190, *d* = 0.37 | 95% *CI*  [-1.14, 0.06],  *t*(143) = 1.78,  *p* = .156, *d* = 0.34 | - | 95% *CI*  [0.53, 1.56], *t*(89.44) = 4.02,  *p* < .001^h^, *d* = 0.79 | 95% *CI*  [0.01, 0.86],  *t*(126) = 2.01,  *p* = .051^3^, *d* = 0.39 |
| **no pref/**  **alone**  **(*n* = 61)** | 95% *CI*  [-1.23, -0.09], *t*(119) = -2.31,  *p* = .048^l^, *d* = 0.42 | 95% *CI*  [-0.79, 0.37],  *t*(119) = -0.71,  *p* = .720, *d* = 0.13 | 95% *CI*  [-0.65, 0.37], *t*(166) = -0.55,  *p* = .711, *d* = 0.09 | 95% *CI*  [-0.23, 1.03],  *t*(97) = 1.25,  *p* = .207, *d* = 0.26 | - | 95% *CI*  [-1.03, -0.19], *t*(107.55) = -2.86,  *p* = .012^i^, *d* = 0.50 |
| **no pref/**  **both**  **(*n* = 90)** | 95% *CI*  [-0.87, -0.15], *t*(148) = -1.39,  *p* = .134, *d* = 0.23 | 95% *CI*  [-0.42, 0.61], *t*(148) = 0.37,  *p* = .720, *d* = 0.06 | 95% *CI*  [-0.28, 0.61], *t*(195) = 0.72,  *p* = .711, *d* = 0.10 | 95% *CI*  [0.13, 1.28],  *t*(126) = 2.41,  *p* = .036^m^, *d* = 0.46 | 95% *CI*  [-0.19, 0.80], *t*(149) = 1.21,  *p* = .460, *d* = 0.20 | - |

**Table S5-III**

*Group Comparisons of the Sociality Ratings from Before and Since Playing PoGo*

*Note*. Below the diagonal: group comparisons from the time period before playing PoGo; above the diagonal: group comparisons from the time period since playing PoGo. *P*-values are based on 1-tailed testing. Superscripts are visualised in Figure S6-III.

# **PoGo Feature Use and Change in Social Functioning/Life Satisfaction**

The majority of participants played PoGo daily (*n* = 346), 35 participants played 6 days a week, 29 participants played 5 days a week, 9 participants played 4 days a week, 8 participants played 3 days a week, 6 participants 2 days a week, and 1 participant one day a week (according to their reports). For the feature use analyses, only the participants who reported daily gameplay were considered as the other cases were flagged as outliers in boxplots.

***Raids***

Raids can be participated in daily and the more difficult the raid is, the more players are needed to succeed. At the time of data collection, only in-person raids were possible. That is, players had to organise themselves and gather at the same location at the same time (for higher-level raids). Players can opt to only do low level raids or play in very popular spots to not have to interact with other players. The majority of the participants reported to raid with other players (*n* = 337) while 94 reported to avoid interaction (3 stated to not know what raids are). The two groups were compared regarding their change scores of the three factors (winsorised) using independent samples *t*-tests (1-tailed). (There are also raid events, generally with more difficult raids, requiring players to form groups for successful participation; 373 participants reported to participate in raid event with 56 not participating (5 stated to not know what raid events are). No analyses were conducted on differences in raid event participation, since the vast majority of the sample participated in these events).

Results showed, players who preferred to raid with others (*M* = 0.55, *SD* = 0.88) had a significantly higher life satisfaction change score than those who prefer to raid alone (*M* = 0.33, *SD* = 0.66), 95% *CI* [0.06, 0.39], *t*(194.76) = 2.67, *p* = .004 (1-tailed), Cohen’s *d* = 0.26. Players who preferred to raid with others (*M* = 0.85, *SD* = 1.08) had a significantly higher social ability change score than those who prefer to raid alone (*M* = 0.31, *SD* = 0.64), 95% *CI* [0.36, 0.71], *t*(255.34) = 6.08, *p* < .001 (1-tailed), Cohen’s *d* = 0.54. Players who preferred to raid with others (*M* = 1.13, *SD* = 1.61) had a significantly higher sociality change score than those who prefer to raid alone (*M* = 0.41, *SD* = 1.04), 95% *CI* [0.45, 0.99], *t*(230.02) = 5.18, *p* < .001 (1-tailed), Cohen’s *d* = 0.48. These results are in line with the hypothesis.

***Community Days***

Once a month, a community day (which stretches over a 3h period) takes place and 314 participants reported to play community days together with others while 110 generally play these events alone. There are (rarely) task-based events where players have to fulfil certain tasks to obtain a specific Pokemon. These events do not require social interaction and 206 players reported to play also these events with others while 220 said they played alone; seven reported to not participate in those events, and one reported to not know what it is. The two groups (alone vs with others) were compared regarding their change scores of the three factors (winsorised) using independent samples *t*-tests (1-tailed).

Results showed, players who preferred to spend community days with others (*M* = 0.56, *SD* = 0.89) had a significantly higher life satisfaction change score than those who preferred to play alone (*M* = 0.38, *SD* = 0.72), 95% *CI* [-0.01, 0.34], *t*(233.26) = 2.09, *p* = .019 (1-tailed), Cohen’s *d* = 0.21. Players who preferred to play with others (*M* = 0.88, *SD* = 1.07) had a significantly higher social ability change score than those who preferred to play alone (*M* = 0.33, *SD* = 0.68), 95% *CI* [0.38, 0.73], *t*(301.49) = 6.23, *p* < .001 (1-tailed), Cohen’s *d* = 0.56. Players who preferred to play with others (*M* = 1.22, *SD* = 1.57) have a significantly higher sociality change score than those who preferred to play alone (*M* = 0.31, *SD* = 1.00), 95% *CI* [0.65, 1.16], *t*(302.32) = 6.97, *p* < .001 (1-tailed), Cohen’s *d* = 0.63.

***Field Research Events***

There are (rarely) task-based events where players have to fulfil certain tasks to obtain a specific Pokemon. These events do not require social interaction and 206 players reported to play also these events with others while 220 said they played alone; seven reported to not participate in those events, and one reported to not know what it is. The two groups (alone vs with others) were compared regarding their change scores of the three factors (winsorised) using independent samples *t*-tests (1-tailed).

Results showed, players who preferred play research events with others (*M* = 0.63, *SD* = 0.95) had a significantly higher life satisfaction change score than those who preferred to play alone (*M* = 0.39, *SD* = 0.74), 95% *CI* [0.08, 0.40], *t*(387.51) = 2.90, *p* = .002 (1-tailed), Cohen’s *d* = 0.28. Players who preferred to spend research events with others (*M* = 0.94, *SD* = 1.18) had a significantly higher social ability change score than those who preferred to play alone (*M* = 0.56, *SD* = 0.89), 95% *CI* [0.18, 0.58], *t*(373.29) = 3.77, *p* < .001 (1-tailed), Cohen’s *d* = 0.37. Players who preferred to play with others (*M* = 1.36, *SD* = 1.65) had a significantly higher sociality change score than those who preferred to play alone (*M* = 0.57, *SD* = 1.25), 95% *CI* [0.51, 1.07], *t*(382.68) = 5.53, *p* < .001 (1-tailed), Cohen’s *d* = 0.54..

***Trading, PvP, and Number of Weekly Raids***

The main social features of the game at the time of data collection were trading, raiding, and PvP (‘How many times per week do you […] in person?’). These features were entered in a linear regression as predictors of the change scores to see which feature has the most benefit. For this purpose, only participants were entered into the analyses who stated to use the features and/or used them socially (*n* = 212). The data were winsorised due to presence of outliers applying the upper and lower boundary limit according to the 25% and 75% Tukey Hinges percentiles.

Results from the regression models showed that the number of conducted raids per week with other PoGo players was a significant predictor of the sociality change score, *F*(1, 210) = 16.04, *p* < .001, *r*^2^ = .07. The resulting equation was: change score in sociality = .54 + (.10 * raids/week). The other predictors included in the models (trading and PvP) were not significant. The number of conducted raids per week with other PoGo players was also a significant predictor of the social ability change score, *F*(1, 210) = 10.95, *p* = .001, *r*^2^ = .05. The resulting equation was: chance score in social ability = .46 + (.06 * raids/week). The other predictors included in the models (trading and PvP) were not significant. None of the entered predictors of the life satisfaction change score were significant.

Results from the feature use analyses showed that carrying out activities with others (raiding and participation in community days and research events) led to higher change scores of life satisfaction, sociality, and social ability than solitude game-play. This raises the question of whether more is more, i.e., are the effects even greater the more players use the features socially? Results showed that out of the social features tested here (trading, raiding, and PvP), only raids could predict the change scores of sociality and social ability; life satisfaction was not associated with the number of raids players conducted socially. That is, the number of raids conducted with others predicted to an extent the increase in sociality and social ability with 7% and 5% of explained variance, respectively. These analyses only included players who stated to use the features and to do so socially; the frequency of social use of the features was entered into the analyses. In sum, whereas the results from the categorical analyses have shown that using a feature socially vs using it in solitude also positively affects life satisfaction, the dimensional analyses (i.e., frequency of the feature use) showed no modulating effect on life satisfaction. However, the frequency of conducting raids with others can modulate the change scores of sociality and social ability. That is, as long as features are being socially enjoyed, benefits for life satisfaction and social functioning will likely be perceived.

# **PoGo and Mental Health (Hypothesis 4)**

Conform with the expected effect of mental disorders on life satisfaction and social functioning, the diagnosis group had significantly lower means on the three factors for both periods before and since playing the game than the group without reported diagnoses, *t*’s(422) = 2.26-8.58, *p*’s ≤ .012.

Group comparisons on the change scores of the three factors were conducted between participants who self-reported to have a formal diagnosis of a mental disorder and such that reported to not have one to examine whether individuals with mental health problems benefit more from playing the game than individuals without a diagnosis. Participants’ change scores of those who self-reported to have a formal diagnosis of a mental disorder (*n* = 90) to those who reported to not have a diagnosis (*n* = 334) on the three factors were compared with independent samples *t*-tests (1-tailed). The data were winsorised due to presence of outliers applying the upper and lower boundary limit according to the 25% and 75% Tukey Hinges percentiles per factor split by group.

Results from the independent samples *t*-tests showed that participants who self-reported to have a formal diagnosis of a mental disorder had a significantly higher change score of life satisfaction (*M* = 0.79, *SD* = 1.04) than those who reported to not have a diagnosis (*M* = 0.42, *SD* = .79), 95% *CI* [0.13, 0.60], *t*(422) = 3.12, *p* = .001 (1-tailed), Cohen’s *d* = 0.44. Participants who self-reported to have a formal diagnosis of a mental disorder had a significantly higher change score of sociality (*M* = 1.31, *SD* = 1.87) than those who reported to not have a diagnosis (*M* = 0.85, *SD* = 1.42), 95% *CI* [0.06, 0.52], *t*(422) = 2.19, *p* = .015 (1-tailed), Cohen’s *d* = 0.22. There was a trend towards a significantly higher social ability change score in those with a self-reported diagnosis of a mental disorder (*M* = 0.68, *SD* = 0.95) and those without (*M* = 0.91, *SD* = 1.30), 95% *CI* [0.05, 0.88], *t*(422) = 1.58, *p* = .059 (1-tailed), Cohen’s *d* = 0.30.

It was expected that levels of depression, trait anxiety, and autism-like traits across the sample are positively associated with the changes in social functioning and life satisfaction. The questionnaires AQ, BDI, STAI were scored according to the individual questionnaire instructions with respective items recoded. Spearman correlations (1-tailed) were conducted between BDI, STAI, and AQ and the change scores from the three factors of life satisfaction, sociality, and social ability to see whether symptom severity is associated with the assumed effects of playing the game. The data were winsorised due to presence of outliers applying the upper and lower boundary limit according to the 25% and 75% Tukey Hinges percentiles for the participants who filled out the AQ (*n* = 337), the BDI (*n* = 254), and the TAI (*n* = 240). To confirm participants’ responses, correlations were conducted with the AQ, BDI, TAI scores and the ratings on the three factors since playing the game where negative associations were expected, i.e., the more severe the symptoms, the more negative the life satisfaction and social functioning.

Against the hypothesis, there were no significant correlations between AQ scores and the change in life satisfaction (*r* = .10, *p* = .081, 1-tailed), change in social ability (*r* = .11, *p* = .081, 1-tailed), or change in sociality (*r* = .09, *p* = .081, 1-tailed). (Although the correlations were trending towards significance, the correlation coefficients’ magnitude is negligible). There was no significant correlation between BDI scores and the change in life satisfaction (*r* = .01, *p* = .736, 1-tailed), and change in social ability (*r* = .02, *p* = .736, 1-tailed), but a very small significant correlation with change in sociality (*r* = .18, *p* = .006, 1-tailed). There was no significant correlation between TAI scores and change in life satisfaction (*r* = .09, *p* = .158, 1-tailed) and change in social ability (*r* = .07, *p* = .158, 1-tailed), but a small significant correlation with change in sociality (*r* = .23, *p* < .001, 1-tailed); see Figure S7-III. The significant correlations between the BDI and TAI with the sociality changes scores are in line with the hypothesis. It is noteworthy that these correlations results were positive, i.e., the greater the symptom severity, the greater the change sociality, while the correlations of life satisfaction, social ability, and sociality since playing the game with the three symptom questionnaires were negative (*p*’s < .001, 1-tailed), i.e., the greater the symptom severity, the lower life satisfaction, social ability, and sociality (Table S6-III).

**Figure S7-III**

*Correlation Between TAI Scores and Change in Sociality*


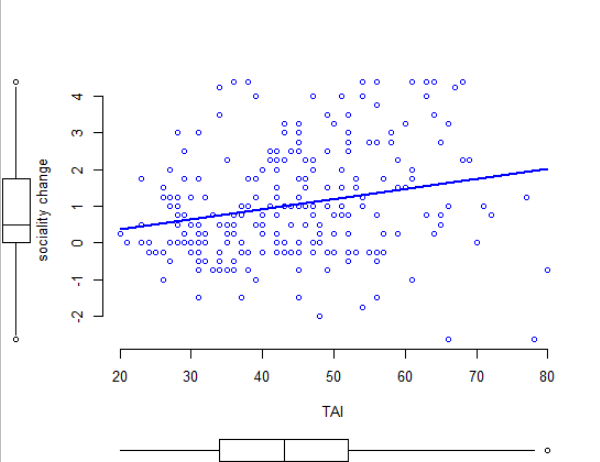


**Table S6-III**

*Spearman’s Rank Correlations Between the Symptom Questionnaires and Life Satisfaction, Social Ability and Sociality*

|  | **AQ**  (*n* = 337) | **BDI**  (*n* = 254) | **TAI**  (*n* = 240) |
| --- | --- | --- | --- |
| **life satisfaction** | *r* = -.377*** | *r* = -.590*** | *r* = -.690*** |
| **social ability** | *r* = -.567*** | *r* = -.292*** | *r* = -.326*** |
| **sociality** | *r* = -.374*** | *r* = -.289*** | *r* = -.280*** |

*Note*. The correlations are based on the ratings since playing PoGo, but the correlation coefficients were almost identical for the ratings before playing PoGo with the questionnaires and are thus not reported here. AQ = Autism Quotient. BDI = Beck’s Depression Inventory. TAI = Trait Anxiety Inventory. ****p* < .001 (1-tailed).

# **References**

Hayes, A. F. (2015). An Index and Test of Linear Moderated Mediation. *Multivariate Behavioral Research*, *50*(1). https://doi.org/10.1080/00273171.2014.962683

Koivisto, J., Gürkan, B. G., Malik, A., & Hamari, J. (2019). Getting Healthy by Catching Them All: A Study on the Relationship between Player Orientations and Perceived Health Benefits in an Augmented Reality Game. *Proceedings of the 52nd Hawaii International Conference on System Sciences*, 1779–1788.

Ruiz-Ariza, A., Casuso, R. A., Suarez-Manzano, S., & Martínez-López, E. J. (2018). Effect of augmented reality game Pokémon GO on cognitive performance and emotional intelligence in adolescent young. *Computers and Education*, *116*, 49–63. https://doi.org/10.1016/j.compedu.2017.09.002

Watanabe, K., Kawakami, N., Imamura, K., Inoue, A., Shimazu, A., Yoshikawa, T., … Tsutsumi, A. (2017). Pokémon GO and psychological distress, physical complaints, and work performance among adult workers: A retrospective cohort study. *Scientific Reports*, *7*(1). https://doi.org/10.1038/s41598-017-11176-2

Yang, C., & Liu, D. (2017). Motives Matter: Motives for Playing Pokémon Go and Implications for Well-Being. *Cyberpsychology, Behavior, and Social Networking*, *20*(1), 52–57. https://doi.org/10.1089/cyber.2016.0562
